# *Arabidopsis* BREVIPEDICELLUS Interacts with the SWI2/SNF2 Chromatin Remodeling ATPase BRAHMA to Regulate *KNAT2* and *KNAT6* Expression in Control of Inflorescence Architecture

**DOI:** 10.1371/journal.pgen.1005125

**Published:** 2015-03-30

**Authors:** Minglei Zhao, Songguang Yang, Chia-Yang Chen, Chenlong Li, Wei Shan, Wangjin Lu, Yuhai Cui, Xuncheng Liu, Keqiang Wu

**Affiliations:** 1 Key Laboratory of South China Agricultural Plant Molecular Analysis and Genetic Improvement, South China Botanical Garden, Chinese Academy of Sciences, Guangzhou, China; 2 University of Chinese Academy of Sciences, Beijing, China; 3 Institute of Plant Biology, National Taiwan University, Taipei, Taiwan; 4 Southern Crop Protection and Food Research Centre, Agriculture and Agri-Food Canada, Ontario, Canada; 5 Department of Biology, Western University, London, Ontario, Canada; 6 State Key Laboratory for Conservation and Utilization of Subtropical Agro-bioresources/ Guangdong Key Laboratory for Postharvest Science, College of Horticultural Science, South China Agricultural University, Guangzhou, China; University of California Riverside, UNITED STATES

## Abstract

*BREVIPEDICELLUS* (*BP* or *KNAT1*), a class-I KNOTTED1-like homeobox (*KNOX*) transcription factor in *Arabidopsis thaliana*, contributes to shaping the normal inflorescence architecture through negatively regulating other two class-I *KNOX* genes, *KNAT2* and *KNAT6*. However, the molecular mechanism of BP-mediated transcription regulation remains unclear. In this study, we showed that BP directly interacts with the SWI2/SNF2 chromatin remodeling ATPase BRAHMA (BRM) both *in vitro* and *in vivo*. Loss-of-function BRM mutants displayed inflorescence architecture defects, with clustered inflorescences, horizontally orientated pedicels, and short pedicels and internodes, a phenotype similar to the *bp* mutants. Furthermore, the transcript levels of *KNAT2* and *KNAT6* were elevated in *brm-3*, *bp-9* and *brm-3 bp-9* double mutants. Increased histone H3 lysine 4 tri-methylation (H3K4me3) levels were detected in *brm-3*, *bp-9* and *brm-3 bp-9* double mutants. Moreover, BRM and BP co-target to *KNAT2* and *KNAT6* genes, and BP is required for the binding of BRM to KNAT2 and KNAT6. Taken together, our results indicate that BP interacts with the chromatin remodeling factor BRM to regulate the expression of *KNAT2* and *KNAT6* in control of inflorescence architecture.

## Author Summary

*BP* is a class-I *KNOX* transcription factor that controls normal inflorescence architecture development by repressing the expression of two *KNOX* genes, *KNAT2* and *KNAT6*. In this study, we showed that *Arabidopsis* BP directly interacts with the SWI2/SNF2 chromatin remodeling ATPase BRM. *brm* and *bp* mutants displayed similar inflorescence architecture defects, with clustered inflorescences, horizontally orientated pedicels, and short pedicels and internodes. Furthermore, BP and BRM co-target to *KNAT2* and *KNAT6* genes and repress their expression. This work reveals a new regulatory mechanism that BP associates with BRM in control of inflorescence architecture development.

## Introduction

In flowering plants, internode patterning and pedicel characteristics are two important determinants of inflorescence architecture, which is highly diversified among flowering plant species [1,2]. Inflorescence architecture results from the activity of the shoot apical meristem (SAM), a cluster of pleuripotent stem cells located at the apex of the primary shoot. In *Arabidopsis*, determining the SAM function is mainly controlled by overlapping activities of two protein family members, the class-I KNOTTED1-like homeobox (KNOX) transcription factor subfamily and the BELL1-like (BELL) transcription factor subfamily. Both KNOX and BELL proteins belong to the three-amino-acid loop extension (TALE) homeodomain superfamily and are able to form heterodimers in determining meristem maintenance [1,2,3,4,5,6,7].

The class-I *KNOX* family contains four members, *SHOOT MERISTEMLESS* (*STM*), *BREVIPEDICELLUS* (*BP*, also called *KNAT1*), *KNAT2*, and *KNAT6* [8]. *STM* is required for the initiation of SAM during embryogenesis and maintenance of proliferation of the cells in SAM [2,9]. *BP*, together with *STM*, contributes to SAM maintenance as loss of function of *BP* reduces the residual meristematic activity of the weak allele *stm-2* [10]. Furthermore, mutations of *BP* in *Arabidopsis* cause severe inflorescence architecture defects, with downward-pointing pedicels, short and abnormal internodes with pronounced node bending [1,2], suggesting that *BP* may play crucial roles in inflorescence architecture development. Further studies showed that *PENNYWISE* (*PNY*), a member of the *BELL* subfamily, could physically interact with BP [4,6,11]. *bp pny* double mutants showed a synergistic phenotype of extremely short internodes interspersed with long internodes and increased branching, suggesting that BP-PNY complex is essential for proper inflorescence architecture development. Moreover, a genetic study showed that inactivation of both *KNAT2* and *KNAT6* could rescue inflorescence architecture defects caused by the *bp* or *pny* single mutation [12]. Increased expression of *KNAT2* and *KNAT6* was detected in *bp* and *pny* mutants, indicating that *BP* and *PNY* may restrict *KNAT2* and *KNAT6* expression to promote correct inflorescence architecture development. Taken together, these studies revealed that the BP-PNY complex regulates inflorescence architecture development mainly by repressing the expression of *KNAT2* and *KNAT6*. However, the molecular mechanism of BP-mediated transcription regulation remains largely unknown.

In eukaryotic cells, gene activity is controlled not only by DNA but also by epigenetic marks. Epigenetic changes involve the modification of DNA activity by methylation, histone modification, and chromatin remodeling [13,14,15,16]. ATP-dependent chromatin remodeling factors use the energy derived from ATP hydrolysis to change the interaction between histone octamer and DNA, and alter the accessibility of genomic regions to transcription factors or the general transcriptional machinery in the context of chromatin [17,18]. BRAHMA (BRM), a member of SWI/SNF ATPases, plays an essential role in reprogramming of transcription in vegetative, embryonic and reproductive plant development in *Arabidopsis* [19,20,21,22]. Mutation 27of *BRM* in *Arabidopsis* causes many morphological defects, such as reduced plant sizes with short roots and small leaves, floral homeotic defects, and earlier flowering [23,24,25]. More recently, BRM was shown to interact with *LEAFY* and *SEPALLATA3*, two key transcription factors involved in controlling floral organ identity by regulating *APETALA3* (*AP3*) and *AGAMOUS* (*AG*) expression [21]. Furthermore, BRM associates with the transcription factor TCP4 in regulation of leaf maturation by modulating the cytokine responsive gene expression [26]. In addition, an interactome screen revealed that BRM interacts with a larger subset of transcription factors, including MYB, bHLH and zinc finger proteins [26]. Collectively, these findings suggest that the SWI/SNF ATPase BRM may act together with different transcription factors in modulating gene expression in plant development processes.

In present work, we demonstrated a direct protein-protein interaction between BRM and BP both *in vitro* and *in vivo*. Furthermore, BRM and BP co-repressed *KNAT2* and *KNAT6* expression in control of inflorescence architecture development.

## Results

### BRM Interacts with BP *In Vitro* and *In Vivo*

**Fig 1:**
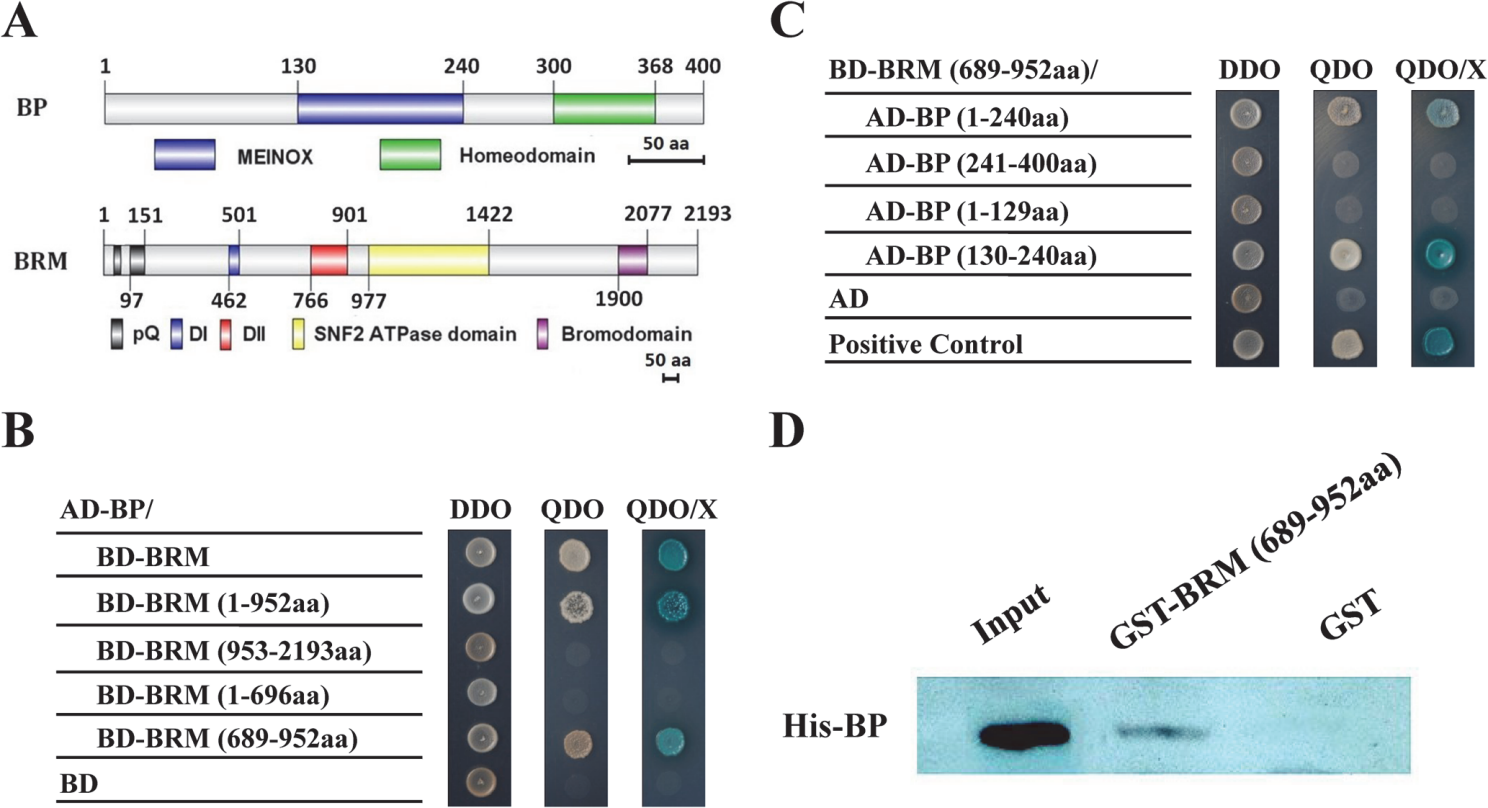
BP interacts with BRM in yeast two-hybrid and *in vitro* pull-down assays. (A) Schematic structures of BP and BRM protein domains. (B,C) Different BRM and BP deletion constructs were cotransformed into the yeast cells GOLD Y2H and plated in DDO. The transformants were also plated on QDO to test for possible interaction. DDO, SD/–Leu/–Trp. QDO, SD/-Leu/-Trp/-His/-Ade. X, x-a-gal. (D) GST-BRM (689-952aa) or GST was incubated with His-BP and His resin, and the bounded proteins were then detected by western blotting using an anti-His antibody. Equal amounts of input His-BP protein were used for pull-down assays.

To identify the interaction proteins of BRM, we performed a yeast two-hybrid library screening. BP was identified as a candidate BRM-interacting partner. Yeast cells co-transformed with AD-BRM (full-length of BRM fused to pGAKT7) and BD-BP (full-length of BP fused to pGBKT7) could grow on selective medium QDO (synthetic medium lacking tryptophan, leucine, histidine and adenine) (Fig. 1A-C), indicating that BRM could directly interact with BP in yeast. Further deletion analysis showed that the DII domain of BRM (amino acids 689–952) and the MEINOX domain (amino acids 130–240) of BP (Fig. 1A-C) were responsible for their interaction. We further detected the interaction between BRM and BP by pull-down assays. Purified BRM (amino acids 689–952)-His was pulled down by GST-BP proteins (Fig. 1D), confirming that BRM physically interacts with BP *in vitro*.

**Fig 2:**
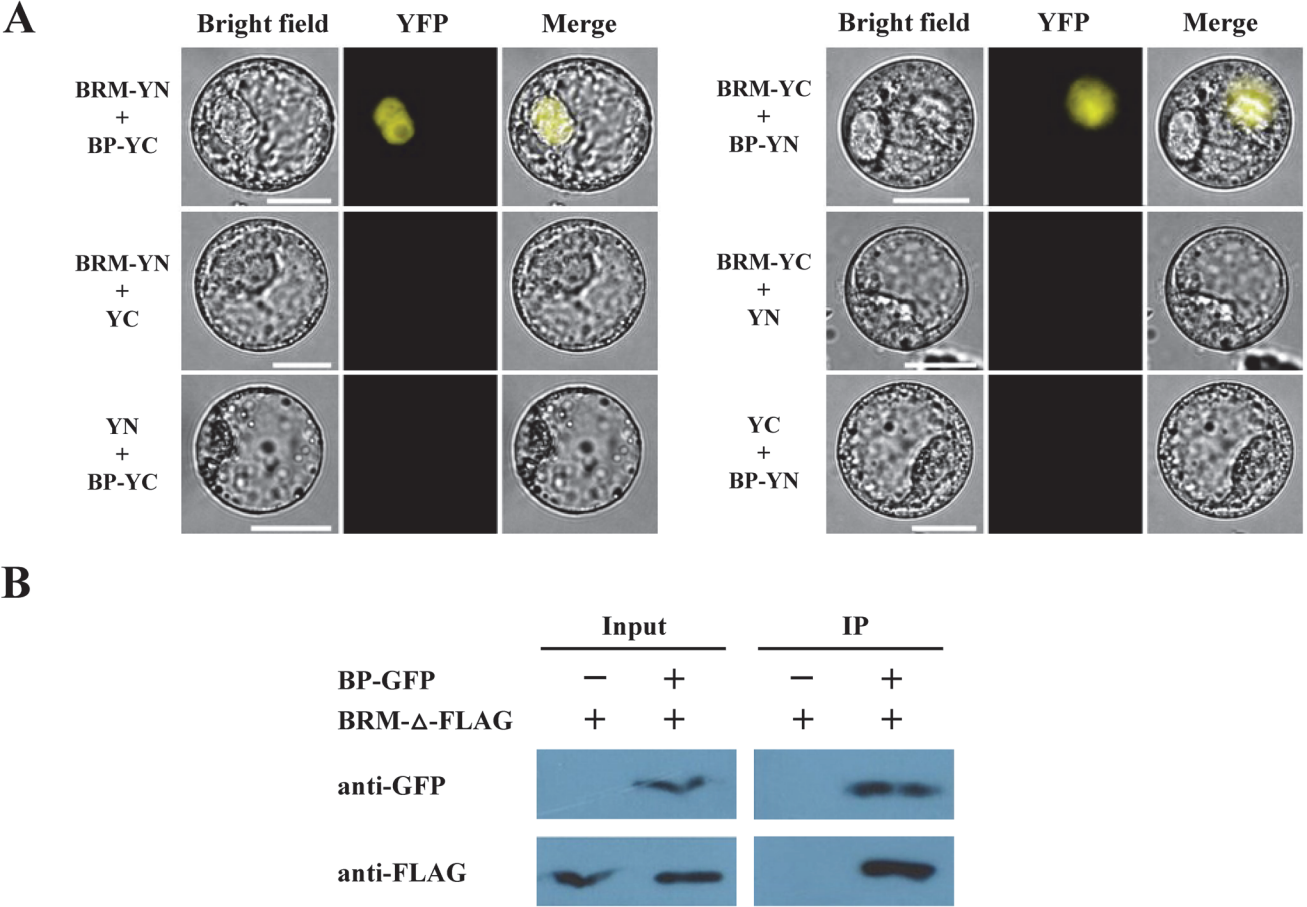
BRM interacts with BP *in vivo* detected by BiFC and Co-IP assays. (A) Full length of BRM and BP fused with the C terminus (YC) or the N terminus (YN) of YFP were co-transformed into tobacco cells. As a negative control, BRM and BP fused with YC or YN and empty vectors were also cotransformed into tobacco cells. (B) The amino acids 689–952 of BRM fused with three FLAG tags (BRM-Δ-FLAG), and the full length of BP was fused with a GFP tag. These constructs were co-transformed into tobacco cells by *Agrobacterium* mediated infiltration assays. Transiently expressed BP-GFP and BRM-Δ-FLAG was immunoprecipitated with an anti-GFP antibody, and then detected by western-blotting assay with an anti-Flag antibody.

The interaction of BRM and BP was further examined *in vivo* by bimolecular fluorescence complementation (BiFC) and co-immunoprecipitation (Co-IP) assays. For the BiFC assay, BRM and BP were fused to the YN vector pUC-pSPYNE or the YC vector pUC-pSPYCE [27]. The constructs were co-delivered into tobacco *Bright Yellow 2* (BY-2) suspension cells by polyethylene glycol (PEG) mediated transformation. As shown in Fig. 2A, BRM interacted with BP in BiFC assays. Among the cells observed, about 10% cells showed positive signals and similar results were obtained in four different experiments. For the Co-IP assay, we transiently expressed BRM and BP proteins in tobacco (*Nicotiana benthamiana*) [14]. As the full length BRM protein could not be well expressed in tobacco cells, we made a construct with the DII domain (amino acids 689–952) of BRM fused with three FLAG tags (BRM-Δ-FLAG). The full length of BP was fused with a GFP tag (BP-GFP). These constructs were co-transformed into tobacco epidermal cells by *Agrobacterium*-mediated infiltration assays. We showed that BRM-Δ-FLAG protein was co-immunoprecipitated by BP-GFP (Fig. 2B). Taken together, these data indicate that BRM interacts with BP both *in vitro* and *in vivo*.

### BRM Is Required for the Inflorescence Development

Previous studies indicated that *BP* is strongly expressed in inflorescences including pedicels and internodes [1]. GUS-staining analyses with *pBRM*:*GUS* plants showed that *BRM* is also expressed in the florescence in *Arabidopsis* (S1 Fig.). Furthermore, expression patterns from the public *Arabidopsis* microarray databases (http://www.bar.utoronto.ca/efp/cgi-bin/efpWeb.cgi) revealed that both *BRM* and *BP* are expressed in shoot apex, stems and internodes in *Arabidopsis* (S2 Fig.). These findings suggested an overlapping expression pattern of *BP* and *BRM* in the inflorescences.

**Fig 3:**
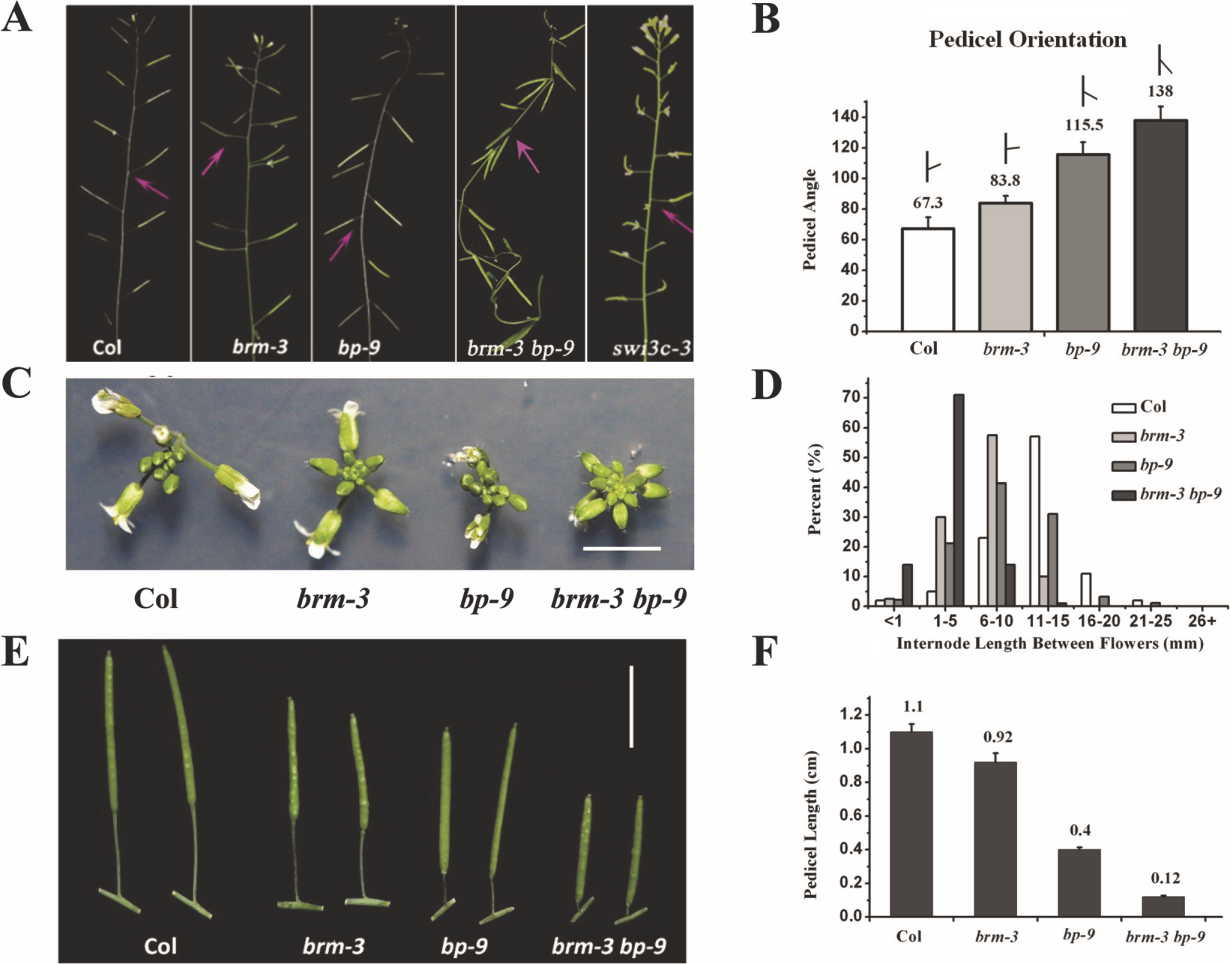
Inflorescence patterns of *brm-3*, *bp-9*, and *brm-3 bp-9* double mutants. (A) Phenotypes of *brm-3*, *bp-9* and *brm-3 bp-9* double mutants in pedicel orientation. The arrows indicate the typical pedicel orientation of the mutants. 35-day-old plants were used for phenotype observation. (B) Quantitative analysis of the pedicel orientation in Col, *brm-3*, *bp-9*, and *brm-3 bp-9* plants (N≥100). (C) Top view of inflorescence in Col, *brm-3*, *bp-9*, and *brm-3 bp-9* plants (bar = 0.5 cm). (D) Distribution of the internode length between two successive siliques. Ten internodes between the 1st and 11th siliques were analyzed. (E) Phenotype of pedicle elongation of the mature siliques in Col, *brm-3*, *bp-9*, and *brm-3 bp-9* plants (bar = 1 cm). 35-day-old plants were used for phenotype observation. (F) Quantitative analysis of the pedicle length of mature siliques in 35-day-old Col, *brm-3*, *bp-9*, and *brm-3 bp-9* plants.

To study the genetic interaction of *BRM* and *BP*, several *brm* alleles, *brm-1* [19], *brm-3* [28], *brm-4 and brm-5* [29], and the null *bp* allele, *bp-9* [1,2], were analyzed. *bp-9* contains a *dSpm* transposon insertion in the 1^st^ intron of *BP* [6]. The transcript of *BP* was not detected in the *bp-9* mutant (S3 Fig.), confirming that *bp-9* is a null allele. Furthermore, the expression level of *BP* was not significantly altered in *brm-3* compared with wild-type (S3 Fig.), suggesting that *BRM* may not affect *BP* expression in inflorescence. We observed that *brm-3* and *bp-9* plants displayed similar inflorescence architecture defects, with horizontally orientated pedicels (Fig. 3A and 3B), clustered inflorescences (Fig. 3C), shorter internodes and pedicels (Fig. 3D-F) compared to wild-type plants. Similar inflorescence architecture defects were also observed in *brm-1*, *brm-4* and *brm1-5* mutant alleles (S4A–S4D Fig.). Interestingly, loss-of-function mutants of *SWITCH/SUCROSE NONFERMENTING 3C* (*SWI3C*) encoding an interaction partner of BRM [25] also showed inflorescence architecture defects as *bp-9* (Fig. 3A).

The null allele *brm-1* was completely sterile [19]. Therefore, we generated the double mutant by crossing the weak allele *brm-3* with *bp-9*. The *brm-3 bp-9* double mutants displayed more severe inflorescence architecture defects compared with *brm-3* and *bp-9* single mutants, with more compacted inflorescences, shorter internodes and pedicels, downward-oriented siliques (Figs. 3A-F and S5). The *brm-3 bp-9* double mutant showed synergistic interaction in inflorescence architecture development, suggesting that additional factors other than BP likely interact with BRM to regulate the same processes. Previous studies indicated that the BELL subfamily transcription factor PNY interacts with BP and is involved in repression of *KNAT2* and *KNAT6* [12]. It is possible that PNY may also interact with BRM in regulating inflorescence architecture development.

**Fig 4:**
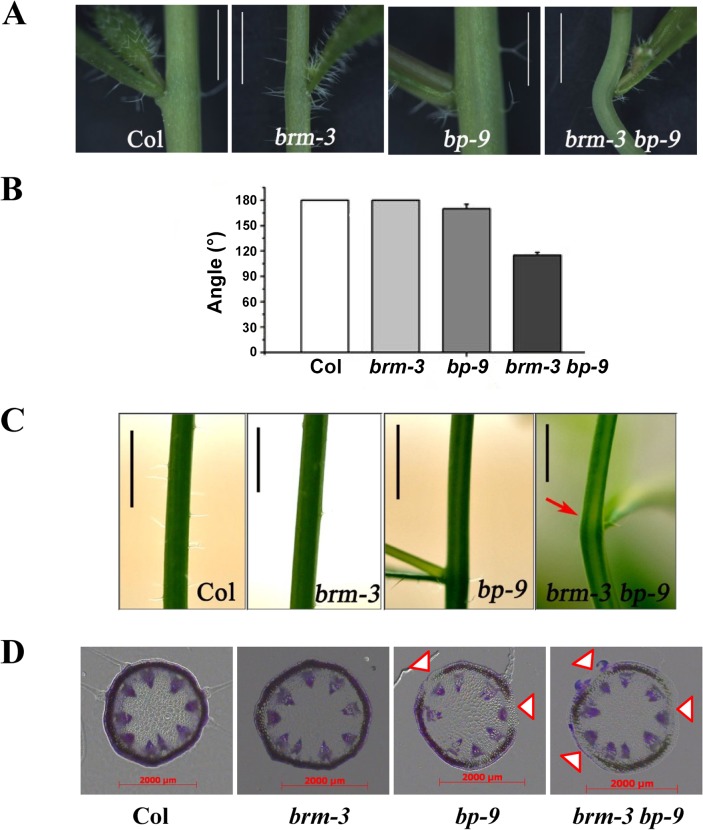
Phenotype of the node in *brm-3*, *bp-9*, and *brm-3 bp-9* mutants. (A) *brm-3 bp-9* showed obvious bend at node (bar = 0.5cm). (B) Quantitative analysis of the angles at the node in Col, *brm-3*, *bp-9*, and *brm-3 bp-9* plants. 50 plants were analyzed. (C) *brm-3 bp-9* displayed chlorenchyma-deficient (as indicated with red arrow) in the stem (bar = 0.5cm). (D) Transverse section of the stem at the nodes of the wild type and mutants. Arrows indicate the regions in which chlorenchyma development is repressed (bar = 2000 μm).

In addition, we also showed that the internodes of *brm-3 bp-9* plants were severely bent (Fig. 4A and 4B). Chlorenchyma are the specialized parenchyma cells, which contain chloroplasts and are distributed in the outer cortex of stems. Bends in stems correlate with a loss of chlorenchyma tissue at the node adjacent to lateral organs [1]. The chlorenchyma density was dramatically reduced in the internodes of *brm-3 bp-9* plants compared with the *bp*-9 single mutant (Fig. 4C and 4D), suggesting an involvement of BRM in control of internode patterns. Taken together, our findings indicate that BRM is required for the inflorescence architecture development in *Arabidopsis*.

### BRM and BP Repress the Transcription of *KNAT2* and *KNAT6*

**Fig 5:**
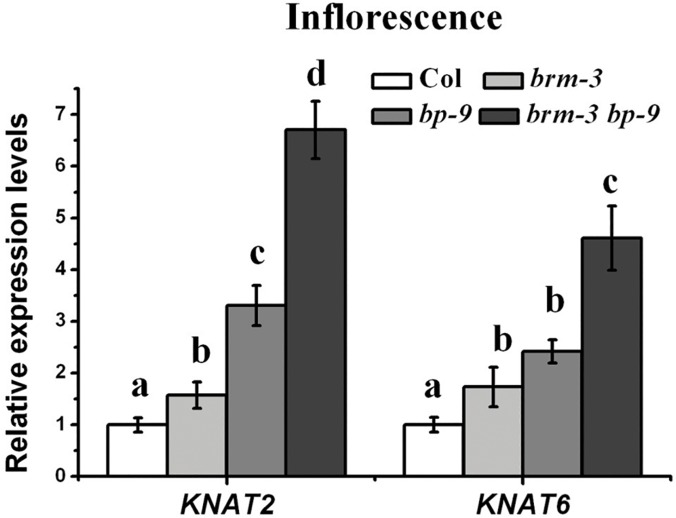
BRM and BP repress *KNAT2* and *KNAT6* expression in inflorescences. qRT-PCR analysis of *KNAT2* and *KNAT6* expression in inflorescences of Col, *brm-3*, *bp-9*, and *brm-3 bp-9* mutants. Data shown are means±SD. *UBQ* was used as an internal control. 35-day-old plants were used for analysis. One-way ANOVA (Tukey-Kramer test) analysis was performed, and statistically significant differences (P < 0.01) were indicated by different lowercase letters (a, b, c, d). Equivalent means have the same letter; different letters indicate statistically significant differences.

Previous studies indicated that inflorescence architecture defects of *bp* mutants are caused by increased expression of two class-I *KNOX* genes, *KNAT2* and *KNAT6* [12]. We further examined the expression levels of *KNAT2* and *KNAT*6 in *brm-3*, *bp-9* and *brm-3 bp-9* plants. The expression levels of *KNAT2* and *KNAT6* in inflorescences of Col, *brm-3*, *bp-9*, *brm-3 bp-9* were analyzed. Compared with wild-type, the expression of *KNAT2* and *KNAT6* was increased in *brm-3*, *bp-9* and *brm-3 bp-9* mutants (Fig. 5). Furthermore, the transcription of *KNAT2* and *KNAT6* was up-regulated in *brm-1* and *brm-4* mutants compared to wild-type plants (S6 Fig.). Much higher expression levels of *KNAT2* and *KNAT6* were detected in the *brm-3 bp-9* double mutant compared to *brm-3* and *bp-9* single mutants (Fig. 5), indicating that *BRM* may function synergistically with *BP* in repression of *KNAT2* and *KNAT6* expression.

### The H3K4me3 Levels of *KNAT2* and *KNAT6* Were Increased in *brm-3*, *bp-9* and *brm-3 bp-9* Plants

**Fig 6:**
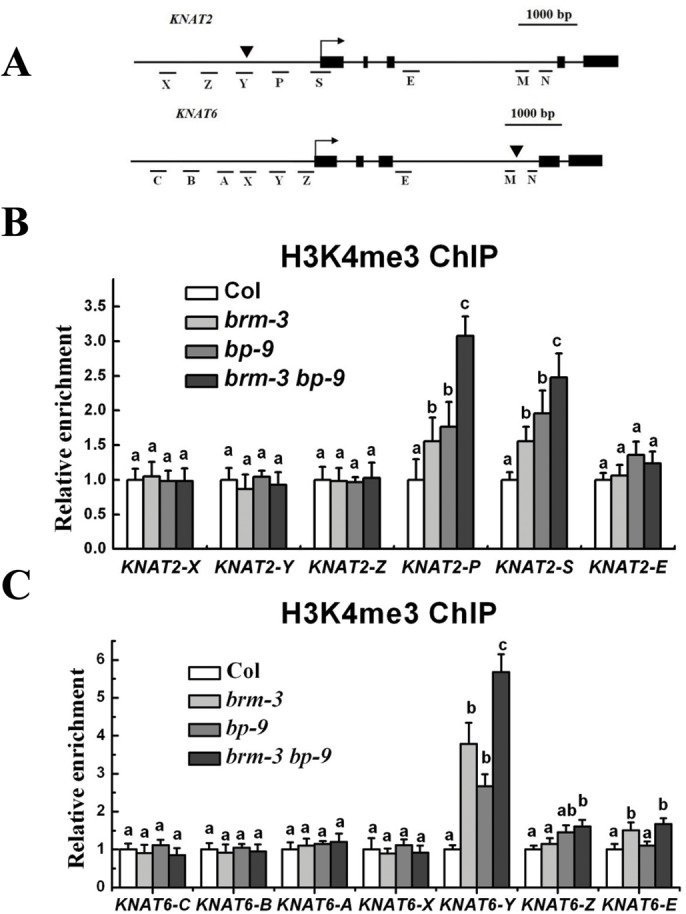
BRM and BP decrease H3K4Me3 levels of *KNAT2* and *KNAT6* in inflorescences. (A) Schematic diagram of *KNAT2 and KNAT6* for ChIP-qPCR analysis. Black boxes indicate the exons. Arrows indicate transcriptional starting sites, whereas black triangles indicate the positions of the TAGC motifs. (B) ChIP-qPCR analysis of relative H3K4me3 levels of *KNAT2* chromatin in Col, *brm-3*, *bp-9*, and *brm-3 bp-9* mutants. (C) ChIP-qPCR analysis of relative H3K4me3 levels of *KNAT6* chromatin in Col, *brm-3*, *bp-9*, and *brm-3 bp-9* mutants. The amounts of DNA after ChIP were quantified and normalized to *TUB2*, the relative enrichment refers to the H3K4me3 enrichment versus the histone H3 occupancy. The values are shown as means±SD. 35-day-old plants were used for analysis. One-way ANOVA (Tukey-Kramer test) analysis was performed, and statistically significant differences (P < 0.01) were indicated by different lowercase letters (a, b, c).

We further determined the levels of the activation marker H3K4me3 and the repression marker H3K27me3 of *KNAT2* and *KNAT6* in *brm-3*, *bp-9* and *brm-3 bp-9* mutants by chromatin immunoprecipitation (ChIP) assays. The relative enrichment of H3K4me3 and H3K27me3 levels was determined by real-time PCR using gene specific primers (Fig. 6A). Increased H3K4me3 levels were detected in both proximal promoter regions (region P of *KNAT2* and region Y of *KNAT6*) and transcription starting sites (region S of *KNAT2* and region Z of *KNAT6*) of *KNAT2* and *KNAT6* in *brm-3*, *bp-9* and *brm-3 bp-9* plants. Elevated H3K4me3 levels were also detected in the intron of *KNAT6* (region E) in *brm-3* and *brm-3 bp-9* mutants compared with wild-type (Fig. 6B and 6C). Increased H3K4me3 levels of *KNAT2* and *KNAT6* observed in *brm-3*, *bp-9* and *brm-3 bp-9* plants are consistent with the up-regulation of these genes in these mutants. Increased expression and H3K4me3 levels of *KNAT2* and *KNAT6* in *brm-3 bp-9* plants were observed compared to *bp-9* plants. The enhanced *brm-3 bp-9* phenotype relative to *bp-9* suggests that additional factors other than BP likely interact with BRM to regulate *KNAT2* and *KNAT6*. By contrast, the H3K27me3 levels of *KNAT2* and *KNAT6* were not significantly altered in *brm-3*, *bp-9* and *brm-3 bp-9* mutants (S7 Fig.).

### BP Binds to *KNAT2* and *KNAT6 In Vitro*

**Fig 7:**
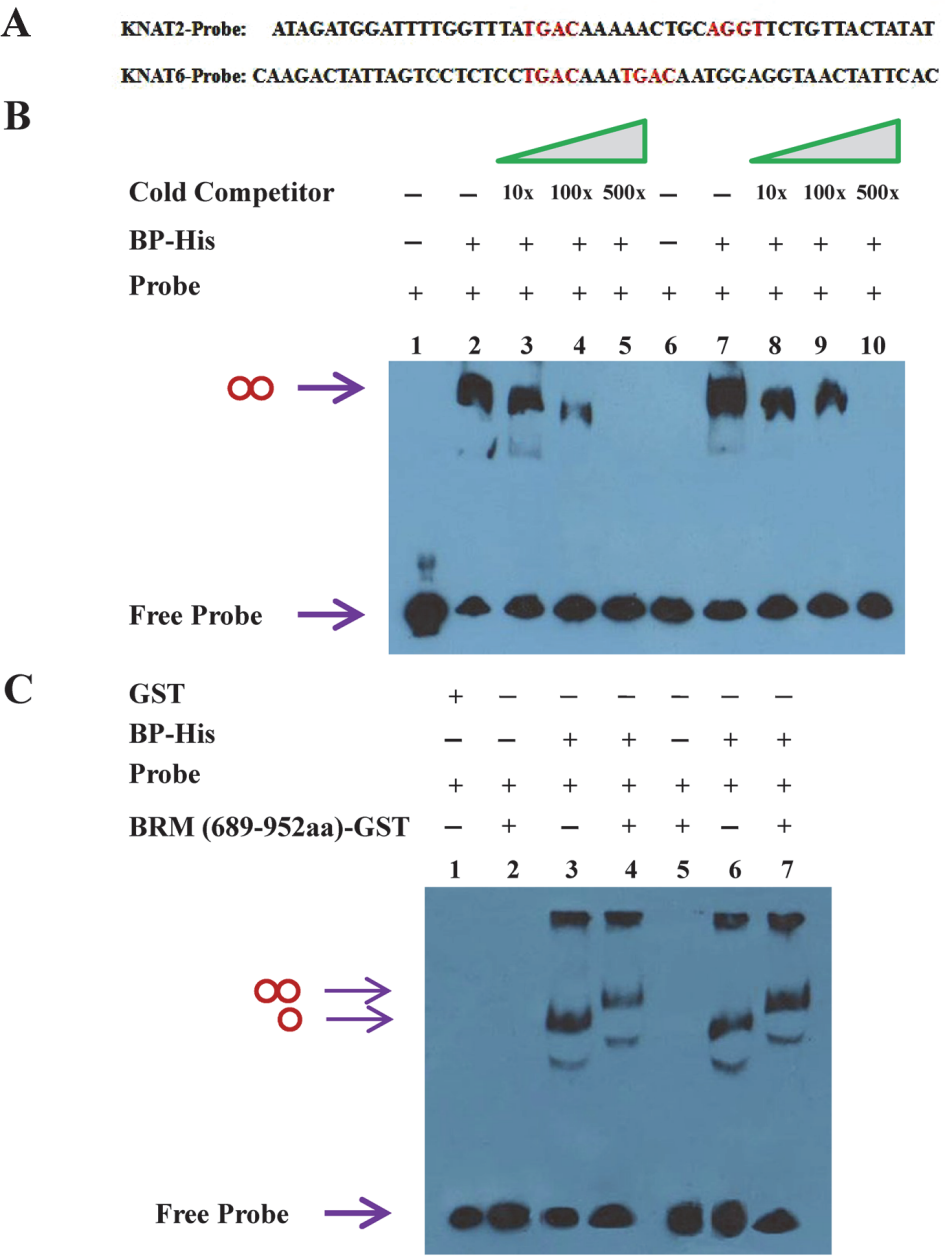
BP binds to *KNAT2* and *KNAT6 in vitro*. (A) Biotin–labeled probe sequence of *KNAT2* (-1039 to -991 bp) and *KNAT6* (4269 to 4319 bp). The core TGAC/AGGT motifs are indicated in red. (B) EMSA assay using purified BP-His fusion protein. Lane 1, 2, 3, 4 and 5 were added with *KNAT2* probe (20 fmol), whereas lane 6, 7, 8, 9 and 10 were added with *KNAT6* probe (20 fmol). 500 ng protein was added in lane 2, 3, 4, 5, 7, 8, 9 and 10, and no protein was added in lane 1 and 6 as negative controls. Non-biotin-labeled probe was added as cold competitor. The double circle indicates binding of the probe id by BP-His. (C) EMSA assay showing that BRM (689-952aa)-GST binds to *KNAT2* and *KNAT6* probes requiring BP. Lane 1 was added with mixed *KNAT2* and *KNAT6* probes, the lane 2, 3 and 4 were added with *KNAT2* probe, and lane 5, 6 and 7 were added with *KNAT6* probe. Lane 1 with GST protein (~500 ng) only was served as a negative control. The single circle indicates the probe is bound by BP-His, and the double circle indicates the probe is bound by BP-BRM (689-952aa) complex.

To examine whether BP protein could directly bind to *KNAT2* and *KNAT6 in vitro*, we performed electrophoretic mobility shift assays (EMSA). The target sequences of KNOX proteins have been identified previously with a core motif of TGAC [30,31]. In maize, the KNOX protein KN1 binds to an intron of *GA2ox1* through a *cis*-regulatory element containing two adjacent TGAC motifs [32]. We identified two TAGC motifs in the promoter of *KNAT2* (-1039 to -991 bp, the Y region as indicated in Fig. 6A) and two TAGC motifs in the third intron of *KNAT6* (4269 to 4319 bp between M and N regions as indicated in Fig. 6A) (Fig. 7A). EMSA assays showed that BP bound strongly to the TAGC motifs of *KNAT2* and *KNAT6* (Fig. 7B). We further showed that the mutated competitor probes could not affect the binding of BP to the TAGC motifs of *KNAT2* and *KNAT6* (S8 Fig.), indicating that BP specifically binds to the TAGC motifs of *KNAT2* and *KNAT6 in vitro* (S8 Fig.).

To determine whether BRM proteins can also directly bind to *KNAT2* and *KNAT6*, purified BRM (689-952aa)-GST protein was incubated with the *KNAT2* and *KNAT6* probes. BRM (689-952aa)-GST alone could not directly bind to *KNAT2* and *KNAT6* (Fig. 7C). When BRM (689-952aa)-GST, BP-His proteins and the *KNAT2* and *KNAT6* probes were incubated together in EMSA assays, two slower shifted bands were detected (Fig. 7C), indicating that BRM may form a complex with BP thus bind to *KNAT2* and *KNAT6 in vitro*.

### BP and BRM Co-Target to *KNAT2* and *KNAT6 In Vivo*

**Fig 8:**
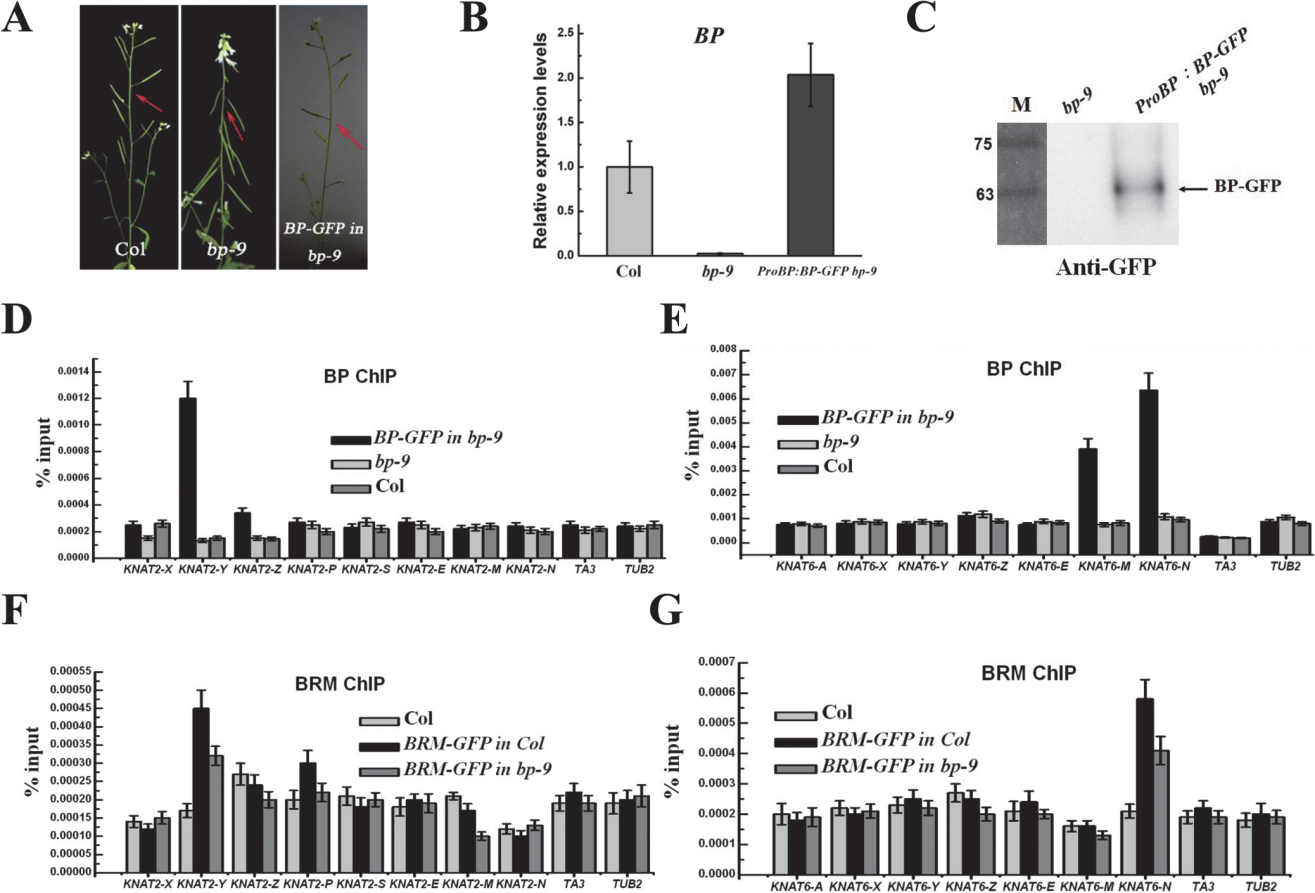
BRM and BP co-target to *KNAT2* and *KNAT6* in inflorescences. (A) Phenotypes of the *ProBP*:*BP-GFP bp-9* (*BP-GFP in bp-9*) transgenic plants. Arrows indicate the typical pedicel orientation of the plants. (B) Detection of the expression level of *BP* in *ProBP*:*BP-GFP bp-9* transgenic plants. (C) Western blotting analysis of BP protein in *ProBP*:*BP-GFP bp-9* transgenic plants. The arrow indicates BP-GFP protein; the numbers indicate the molecular mass in kilodaltons. (D, E) ChIP-qPCR analysis of BP-GFP DNA fragments co-immunoprecipitated with the anti-GFP antibody in *KNAT2* and *KNAT6* chromatin. Relative enrichment was calculated based on IP/input for each sample. *TA3* and *TUB2* were used as negative control. The values are shown as means±SD. (F, G) ChIP-qPCR analysis of BRM-GFP DNA fragments co-immunoprecipitated with the anti-GFP antibody in *KNAT2* and *KNAT6* chromatin. *BRM-GFP* in Col is a transgenic line expressing GFP-tagged BRM in Col background, while *BRM-GFP in bp-9* is a transgenic line expressing GFP-tagged BRM in *bp-9* mutants. Relative enrichment was calculated based on IP/input for each sample. *TA3* and *TUB2* were used as negative control. The values are shown as means±SD.

To study whether *KNAT2* and *KNAT6* are direct targets of BP *in vivo*, ChIP assays were performed using transgenic plants expressing green fluorescent protein (GFP)-Tagged BP driven by the native BP promoter (*ProBP*:*BP-GFP*). Expression of *ProBP*:*BP-GFP* in *bp-9* background fully rescued the inflorescence architecture defects of *bp-9* (Fig. 8A-C), suggesting that BP-GFP is functional *in vivo*. BP strongly bound to the proximal promoter region (Y) of *KNAT2* and the third intron (M and N) of *KNAT6* (Fig. 8D and 8E), indicating that *KNAT2* and *KNAT6* are direct target genes of BP.

We further analyzed whether *KNAT2* and *KNAT6* are also direct targets of BRM *in vivo*. The transgenic plants expressing GFP-tagged BRM driven by the *BRM* native promoter (*ProBRM*:*BRM-GFP*) [33] was used to perform the ChIP assay. *ProBRM*:*BRM-GFP brm-1* and *ProBRM*:*BRM-GFP brm-3* plants were generated by crossing *ProBRM*:*BRM-GFP* plants with *brm-1* and *brm-3* plants, respectively. The growth defects of *brm-1* and *brm-3* were rescued by *ProBRM*:*BRM-GFP*, indicating that BRM-GFP is functional *in vivo* (S9 Fig.). In addition, *ProBRM*:*BRM-GFP bp-9* plants were also generated by crossing *ProBRM*:*BRM-GFP* plants with *bp-9*. Similar to the previous studies [20], we showed that BRM bound to the promoter region of *ABI5*, but not to the control genes, *TA3* and *TUB2* (S10 Fig.).

Similar to BP, BRM also bound to the proximal promoter region (Y) of *KNAT2* and the third intron (N) of *KNAT6* (Fig. 8F and 8G), suggesting that BRM and BP co-target to *KNAT2* and *KNAT6 in vivo*. Compared to *ProBRM*:*BRM-GFP* plants, a decrease of binding of BRM to *KNAT2* and *KNAT6* was observed in *ProBRM*:*BRM-GFP bp-9* plants (Fig. 8F and 8G). Taken together, these analyses suggest that BP is required for the binding of BRM to *KNAT2* and *KNAT6*.

### Removal of *KNAT2* and *KNAT6* Activity Partially Rescues the *brm-3* Phenotype

**Fig 9:**
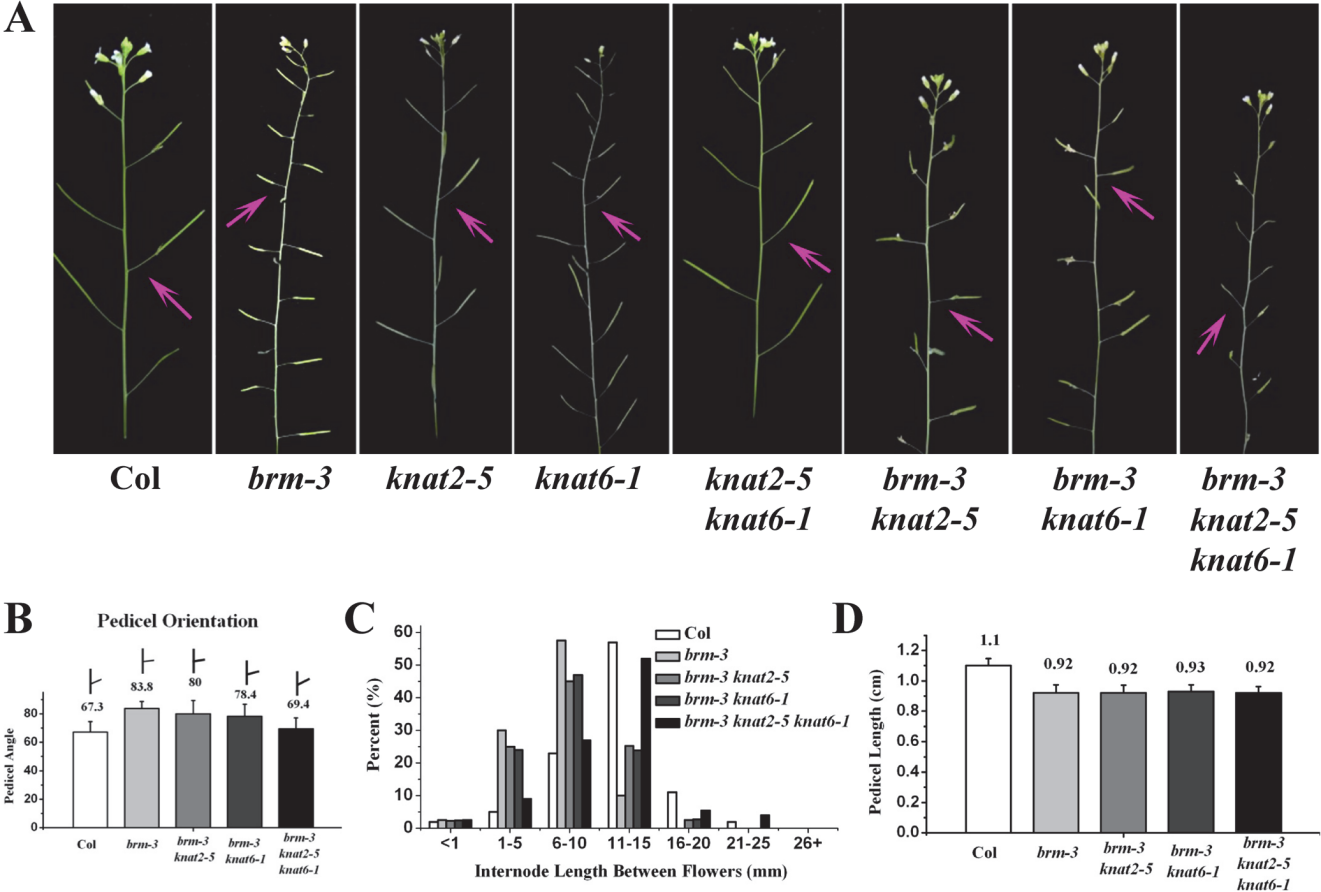
Removal of *KNAT2* and *KNAT6* rescues the *brm-3* phenotype in pedicel orientation and internode length. (A) Phenotypes of *brm-3*, *brm-3 knat2-5*, *brm-3 knat6-1*, and *brm-3 knat2-5 knat6-1* plants in pedicel orientation. The arrows indicate the typical pedicel orientation of the mutants. 35-day-old plants were used for phenotype observation. (B) Quantitative analysis of the pedicel orientation in Col, *brm-3*, *brm-3 knat2-5*, *brm-3 knat6-1*, and *brm-3 knat2-5 knat6-1* plants. (C) Distribution of the internode length between two successive siliques. Ten internodes between the 1st and 11th siliques were analyzed. (D) Quantitative analysis of the pedicle length of mature siliques in 35-day-old Col, *brm-3*, *brm-3 knat2-5*, *brm-3 knat6-1*, and *brm-3 knat2-5 knat6-1* plants.

We further analyzed the genetic interaction of *BRM* with *KNAT2* and *KNAT*6 in inflorescence architecture development. We generated *brm-3 knat2-5* and *brm-3 knat6-1* double mutants as well as *brm-3 knat2-5 knat6-1* triple mutants by genetic crossing *brm-3* with *knat2-5* and *knant6-1* alleles [34]. The pedicel angel, internode and pedicel length were determined in *brm-3*, *brm-3 knat2-5*, *brm-3 knat6-1* and *brm-3 knat2-5 knat6-1* plants. Similar to a previous report [12], no difference was found in the pedicel and internode length of the *knat2-5*, *knat6-1* and *knat2 knat6* mutants compared to wild-type. Compared to *brm-3* plants, a significant decrease of average pedicel angel was found in *brm-3 knat2-5 knat6-1* but not in *brm-3 knat2-5* and *brm-3 knat6-1* plants (Fig. 9A and 9B). Quantitative phenotype analysis showed that *knat2* and *knat6* mutations could fully rescue the pedicel orientation defect of *brm-3*, indicating a requirement of both *KNAT2* and *KNAT6* in control of pedicel orientation. The distribution of internodes along the main inflorescence was also determined. The *brm-3 knat2-5*, *brm-3 knat6-1* and *brm-3 knat2-5 knat6-1* mutants showed longer internodes compared to *brm-3* plants (Fig. 9C). However, removal of both *KNAT2* and *KNAT6* activity could not rescue the pedicel length of *brm-3*, since *brm-3*, *brm-3 knat2-5*, *brm-3 knat6-1* and *brm-3 knat2-5 knat6-1*mutants displayed a similar pedicel length (Fig. 9D). Taken together, our findings suggest that inactivation of *KNAT2* and *KNAT6* partially rescues the *brm-3* phenotype.

## Discussion

### BRM Is Required for Inflorescence Architecture Development

In eukaryotes, the ATP dependent SWI/SNF chromatin remodeling complexes use energy from ATP hydrolysis to alter the interaction between histones and DNA and control accessibility of cis-regulatory DNA regions to transcription machinery [35]. BRM, a member of SWI/SNF ATPases, plays an essential role in reprogramming of transcription in vegetative, embryonic and reproductive development in *Arabidopsis* [19,20,21,22,29]. In present work, we showed that BRM is required for inflorescence architecture development. Loss of function *BRM* mutants display inflorescence architecture defects, with clustered inflorescences and horizontally orientated pedicels. Mutations of *SWI*3C, another *SWI2/SNF2* chromatin remodeling ATPase gene in *Arabidopsis*, also cause a horizontally-pointing pedicel phenotype. BRM was shown to interact with SWI3C and they function in the same protein complex [25]. The similar pedicel orientation defect of *brm* and *swi*3c mutants supports an involvement of the SWI/SNF ATPases chromatin remodeling complex in inflorescence architecture development.

### BRM Represses *KNAT2* and *KNAT6* Expression

The SWI/SNF complex has a co-activator function, catalyzing chromatin remodeling and recruiting activator determinants to gene sequences [36]. Furthermore, SWI/SNF can remodel chromatin resulting in either activation or repression of gene expression [37]. In present work, increased expression of two *KNOX* genes, *KNAT2* and *KNAT6*, was detected in *brm-3*, *brm-1* and *brm-4* plants. EMSA and ChIP experiments showed that *KNAT2* and *KNAT6* are the direct target genes of BRM both *in vitro* and *in vivo*. These findings suggest that BRM may act as a repressor in regulation of *KNAT2* and *KNAT6* expression in *Arabidopsis*. The human BRM was shown to associate with Methyl CpG Binding Protein 2 (MeCP2) *in vivo* and is functionally linked with gene repression [38]. Moreover, a direct association of BRM with the histone demethylase UTX was also reported in *Drosophila melanogaster* [39]. Increasing levels of H3K4me3 in *KNAT2* and *KNAT*6 in *brm-3* indicate that BRM may associate with a histone H3K4 demethylase in repression of gene expression.

Previous studies showed that SWI/SNF ATPases act antagonistically with Polycomb-group (PcG) proteins in gene expression in mammalian [40]. PcG proteins are subunits of two multi-protein complexes, Polycomb Repressive Complex 1 (PRC1) and PRC2 [41,42]. PRC2 catalyses the trimethylation of lysine 27 of histone H3 (H3K27me3) [43,44]. More recently, it was reported that *KNAT2* is repressed by ASYMMETRIC LEAVES 1 (AS1) and AS2 via recruitment of PRC2 [45]. However, the H3K27me3 levels of *KNAT2* and *KNAT6* were not changed in *brm* mutants. Further research is required to investigate the interaction between BRM and PcG proteins in repression of *KNAT2* and *KNAT6*.

### BP Associates with BRM in Regulation of Inflorescence Architecture

A previous study showed that *knat2 knat6 bp* mutants rescue the pedicel orientation and internode length defects of the *bp* mutant [12]. Similarly, we found that introduction of *knat2-5* and *knat6-1* into *brm-3* can also rescue the pedicel orientation and internode length phenotypes of *brm-3*. Increased expression of *KNAT2* and *KNAT6* was found in both *brm* and *bp* mutants. These findings indicate that BRM and BP act upstream of *KNAT2* and *KNAT*6 in regulation of inflorescence architecture. ChIP analysis indicated that BRM and BP co-target to *KNAT2* and *KNAT6* genes, suggesting that BRM and BP directly regulate *KNAT2* and *KNAT6* expression in the inflorescences. Furthermore, *brm-3 bp-9* double mutants displayed more severe inflorescence architecture defects compared with *brm-3* and *bp-9* single mutants, supporting that *BRM* acts synergistically with *BP* in regulation of inflorescence development. *knat2* and *knat6* mutations did not rescue the shorter pedicel phenotype of *brm-3* and *bp* mutants [12], suggesting that other genes are also involved in the control of pedicel growth. KNOX proteins promote shoot apical meristem activity by coordinately regulating cytokinin (CK) and gibberellin (GA) biosynthesis genes [46]. Furthermore, BRM could directly regulate GA and CK responsive genes to promote leaf growth and shoot apical meristem activity [26,47]. Further research is required to identify additional target genes regulated by BRM and BP in promotion of cell proliferation and elongation in *Arabidopsis*.

Accurate initiation of gene transcription requires multiple factors, including transcription cofactors (coactivator or corepressors) and chromatin remodeling factors [13]. *In vitro* studies have shown that transcription factors recruit chromatin remodeling factors and histone modification factors to affect the chromatin status of specific loci [48]. For example, two Jumonji N/C (JmjN/C) domain-containing proteins, ELF6 and REF6, are recruited by their interacted transcription factor BES1 to regulate their co-target genes and coordinate BR responses [49]. Histone deacetylase HDA15 is recruited by its interacted partner PIF3 to repress chlorophyll biosynthetic and photosynthetic genes in etiolated seedlings [50]. In addition, HDA6 and HDA19 are recruited by AS1 and HSL2, respectively, to regulate gene expression involved in leaf and seed development [51,52,53]. More recently, the chromatin remolding factor BBM was shown to interact with transcription factors in yeast two-hybrid assays [26], indicating that BRM may be associated with different transcription factors involved in regulation of gene expression. In present work, we showed that BRM physically interacted with BP both *in vitro* and *in vivo*, suggesting that BP may associate with BRM to regulate gene expression. Furthermore, the binding of BRM to the target genes depended on the presence of BP, indicating that BRM may be recruited by BP through the protein-protein interaction. PNY, a member of the BELL subfamily protein, has been shown to interact with BP physically [6]. In addition, PNY was also shown to play a role in repressing of *KNAT2* and *KNAT6* expression. It remains to be determined whether PNY is also associated with BRM in regulating inflorescence patterning by epigenetic regulation of *KNAT2* and *KNAT6* expression.

## Materials and Methods

### Plant Materials

*brm-1*, *brm-3* (SALK_088462), *brm-4* (WiscDsLox436E9), *brm-5* and *swi*3c-*3* (SAIL_224_B10) were obtained from the Arabidopsis Biological Resource Center (http://www.arabidopsis.org/). *knat6-1* (SALK_047931) and *knat2-5* (SALK_099837) were obtained from Nottingham Arabidopsis Stock Centre (NASC). *bp-9* was kindly provided by Prof. Lin Xu (Shanghai Institute of Plant Physiology and Ecology, Chinese Academy of Sciences).

*ProBP*:*BP-GFP bp-9* transgenic plants were generated by transforming the *ProBP*:*BP-GFP* construct into *bp-9* plants using the floral dip method [54]. The *ProBRM*:*BRM-GFP bp-9* plants were generated by crossing *ProBRM*:*BRM-GFP* plants [33] with *bp-9* plants. All *Arabidopsis* plants were grown in 22°C under long-day (16 h light/8 h dark) conditions.

### Phenotypic Analysis

The pedicel orientation, pedicel length and internode (the stem between two nodes) length between siliques were measured in 35-day-old plants. 10 individual plants were used for quantitative analysis, and 8–10 pedicels were measured for each plant. The minimum age of pedicel selected for analysis is 15 days after flowering. A protractor was used to determine the angle of pedicels. Bend at node was imaged using a stereoscope (ZEISS, SV11). The thin sections of chlorenchyma tissue were prepared with a razor blade and observed under a microscope.

### Quantitative RT-PCR Analysis

Total RNA was isolated from inflorescences (0.15 g) of 35-day-old plants using 1 mL Trizol reagent (Invitrogen). The first strand cDNA synthesis was generated using 2 μg total RNA according to the manufacturer’s instructions of TransScript One-Step gDNA Removal and cDNA Synthesis SuperMix Kit (TransGen, Beijing). 100 ng synthesized cDNA was used as a template to perform quantitative RT-PCR analysis. PCR reactions were performed in the total volume of 20 μL, with 0.5 μL for each primer (10 mm, final concentration 100 nm) and 10 μL for SYBR Green PCR Supermix (Bio-Rad Laboratories) on a ABI7500 Real-Time PCR System (Applied Biosystems). The PCR program included an initial denaturation step at 94°C for 3 min, followed by 40 cycles of 5 s at 94°C and 1 min at 60°C. Each sample was quantified at least triplicate and normalized using *Ubiquitin 10* (*UBQ*) as an internal control. The gene-specific primer pairs for quantitative Real-Time PCR are listed in S1 Table. All PCR reactions were normalized using Ct value corresponding to the reference gene *UBQ*. The relative expression levels of target gene were calculated with formula 2^-ddCt^ [55]. Values represented the average of three biological replicates.

### Yeast Two-Hybrid Assays

Yeast two-hybrid assays were performed as described in the manual of Matchmaker Gold Yeast Two-Hybrid Systems (Clontech). Full length and different deletion coding regions of *BRM* and *BP* were subcloned into pGBKT7 and pGADT7 vectors to construct different bait and prey constructs (primers are listed in S1 Table). Then, different pairs of bait and prey constructs were co-transformed into yeast strain Gold Y2H by PEG, and yeast cells were grown on DDO medium (minimal media double dropouts, SD medium with-Leu/-Trp) for 3 days. Transformed colonies were dropped onto QDO medium (minimal media quadruple dropouts, SD medium with-Leu/-Trp/-Ade/-His) containing 4 mg mL^-1^ X-a-Gal (QDO/ X) to test for possible interactions between BRM and BP according to their growth status.

### *In Vitro* Pull-Down Assays

*In vitro* pull-down assays were performed as described [50]. His-BP recombinant protein was incubated with 30 mL His resin (QIAGEN) in a phosphate buffer (10 mM Na_2_HPO_4_, 10 mM NaH_2_PO_4_, 500 mM NaCl, and 10 mM imidazole) for 2 h at 4°C, the binding reaction was washed three times with the phosphate buffer, and then BRM (689-952aa)-GST or GST was added and incubated for an additional 2 h at 4°C. After washing three times with the phosphate buffer, the pulled-down proteins were eluted by boiling, separated by 10% SDS-PAGE, and detected by western blotting using an anti-His antibody.

### BiFC Assays

For BiFC assays, full length coding regions of BRM and BP were subcloned into YN vector pUC-pSPYNE and the YC vector pUC-pSPYCE, respectively [27]. Then fused YN and YC constructs were transformed into tobacco cells by polyethylene glycol for transient expression [56]. Transfected protoplast cells were imaged using a TCS SP5 confocal spectral microscope imaging system (Leica).

### Co-IP Assays

Co-IP assays were performed as described previously [14]. Two days after infiltration, tobacco (*Nicotiana benthamiana*) leaves were harvested and ground in liquid nitrogen. Proteins were extracted in an extraction buffer (50 mM Tris-HCl, pH 7.4, 150 mM NaCl, 2 mM MgCl_2_, 1 mM DTT, 20% glycerol, and 1% NP-40) containing protease inhibitor cocktail (Roche). Cell debris was pelleted by centrifugation at 14,000g for 20 min. The supernatant was incubated with 30 μL of GFP-Trap A beads (Chromo Tek) at 4°C for 4 h, then the beads were centrifuged and washed six times with a washing buffer (50 mM Tris-HCl, pH 7.4, 150 mM NaCl, 2 mM MgCl_2_, 1mM DTT, 10% glycerol, and 1% NP-40). Proteins were eluted with 40 μL of 2×loading buffer and analyzed by western blotting using anti-GFP (Roche) and anti-Flag antibodies (Life Tein).

### ChIP Assays

ChIP assays were performed as previously described [57]. Chromatin was extracted from the inflorescence tissues (0.3 g) bearing the first 10 siliques of 35-d-old flowering plants, after fixation with formaldehyde, the chromatin was extracted and then sheared to an average length of 500 bp by sonication. The chromatin was immunoprecipitated with specific antibodies including anti-H3K27me3 (Millipore, 07–449), anti-H3K4me3 (Millipore, 07–473), and anti-GFP (Abcam, ab290). The histone H3 occupancy at specific gene loci was analyzed by using an anti-H3 antibody (Millipore 06–775). Equal amount of the sonicated chromatin solution was set aside as the input sample. After cross-linking reversed, the amount of precipitated DNA fragments and input DNA was detected by quantitative Real-Time PCR using specific primers listed in S1 Table. The relative enrichments of various regions of *KNAT2* and *KNAT*6 in *brm-3*, *bp-9* and *brm-3 bp-9* over Col were calculated after normalization to *TUB2*. The percentage of input was calculated by determining 2^-ΔCt^ (= 2^-[Ct(ChIP)-Ct(Input)]^). The exon region of retrotransposon *TA3* [58] was used as negative control.

### EMSAs

In EMSAs, purified recombinant BP-His and BRM (689-952aa)-GST proteins are used. Oligonucleotide probes of *KNAT2* (-1039 to -991 bp) and *KNAT6* (4269 to 4319 bp) sequences were commercially synthesized with 5'-end biotin-labeled as single-stranded DNA (Invitrogen). To generate double-stranded oligonucleotides, equal amounts of complementary single-stranded oligonucleotides were mixed, heated to 95°C for 5 min, and slowly cooled down to 25°C. For a binding reaction, the Light Shift Chemiluminescent EMSA kit (Pierce) was used. For BP-His or BRM (689-952aa)-GST binding, the purified protein is incubated with binding buffer (2.5% glycerol, 5 mM MgCl_2_, 50 ng/μL poly [dI.dC], 0.05% Nonidet P-40) mixed with the labeled probe for 1 h at 4°C in 20 μL reaction volume. For cold competition, the non-labeled probe is added first for 1 h at 4°C followed by the labeled probe added. For BP-His and BRM (689-952aa)-GST interaction complex binding, first purified BP-His and BRM (689-952aa)-GST proteins were incubated together as the GST-pull down assay, then the mixed proteins were used for the EMSA assay. After the binding incubation, the reaction mixture is loaded on a 5% polyacrylamide gel (acrylamide:bisacrylamide, 29:1; Bio-Rad) and run in 0.5×Tris-borate-EDTA buffer at 4°C. The DNA-protein complex was transferred to a Hybond-N+ membrane, and the membrane was cross-linked. Detection was performed according to the manufacturer’s instructions (Pierce).

### Accession Numbers

Sequence data from this article can be found in the Arabidopsis Genome initiative or GenBank/EMBL databases under the following accession numbers: *BRM* (*AT*2G*46020*), *BP* (*AT*4G*08150*), *KNAT2* (*AT*1G*70510*), *KNAT6* (*AT*1G*23380*), *SWI*3C (*AT*1G*21700*), *TUB2* (*AT*5G*62690*), *TA3* (*AT*1G*37110*) and *PNY* (*AT*5G*02030*).

## Supporting Information

S1 Fig Expression patterns of *pBRM*:*GUS* in *Arabidopsis*. (A) GUS staining of *BRM* promoter: *GUS* (*pBRM*:*GUS*) observed in the leaf vascular tissues. (B) GUS staining of *pBRM*:*GUS* observed in inflorescences.
(TIF)

S2 Fig Expression patterns of *BRM* (A) and *BP* (B) from public *Arabidopsis* microarray database (http://www.bar.utoronto.ca/efp/cgi-bin/efpWeb.cgi). Red arrows indicate the expression levels in shoot apex, stems and internodes.
(TIF)

S3 Fig qRT-PCR analysis of expression levels of *BP* in inflorescence of Col, *brm-3* and *bp-9* plants. Data shown are means±SD. *UBQ* was used as an internal control. One-way ANOVA (Tukey-Kramer test) was performed, and statistically significant differences (P < 0.01) are indicated by different lowercase letters (a, b). Equivalent means have the same letter; different letters indicate statistically significant differences.
(TIF)

S4 Fig Inflorescence patterns of *brm-1*, *brm-4* and *brm-5* alleles. (A) Phenotypes of *brm-1*, *brm-4* and *brm-5* mutants. The red color arrows indicate the typical pedicel orientation and internode length of the mutants. (B) Quantitative analysis of the pedicel orientation of *brm-1*, *brm-4* and *brm-5* mutants. (C) Distribution of the internode length between two successive siliques in Col, *brm-1*, *brm-4* and *brm-5* mutants. Ten internodes between the 1st and 11th siliques were analyzed. (D) Quantitative analysis of the pedicle length of mature siliques. 35-day-old plant were analyzed.
(TIF)

S5 Fig The whole plant images of Col, *brm-3*, *bp-9* and *brm-3 bp-9*. (A) 20-d-ld plants of Col, *brm-3*, *bp-9* and *brm-3 bp-9* during vegetative growth. (B) 40-d-old plants of Col, *brm-3*, *bp-9* and *brm-3 bp-9*.
(TIF)

S6 Fig qRT-PCR analysis of *KNAT2* and *KNAT6* expression in Col, *brm-1* and *brm-4* mutants. Data shown are means±SD. *UBQ* was used as an internal control. One-way ANOVA (Tukey-Kramer test) was performed, and statistically significant differences (P < 0.01) are indicated by different lowercase letters (a, b). Equivalent means have the same letter; different letters indicate statistically significant differences.
(TIF)

S7 Fig ChIP analysis of H3K27me3 levels of *KNAT2* and *KNAT6* in *brm-3*, *bp-9* and *brm-3 bp-9* mutants. The amounts of DNA after ChIP were quantified and normalized to *TUB2*. The relative enrichment refers to the H3K27me3 enrichment versus the histone H3 occupancy. The values are shown as means±SD, a single asterisk indicate significant differences from Col by Student’s *t* test (*P < 0.05). 35-day-old plants were used for analysis. The position of the primers are as indicated in Fig. 6A.
(TIF)

S8 Fig EMSA assays with mutated competitor probes. (A) Biotin–labeled mutated probe sequences of *KNAT2* (-1039 to -991 bp) and *KNAT6* (4269 to 4319 bp). The core binding sites were mutated as shown with red underline. (B) EMSA assay using purified BP-His fusion protein. Lane 1, 2, 3 and 4 were added with *KNAT2* probe (20 fmol), whereas lane 5, 6, 7 and 8 were added with *KNAT6* probe (20 fmol). 500 ng of BP-His protein was added in lane 2, 3, 4, 6, 7 and 8, and no protein was added in lane 1 and 5 as negative controls. Mutant *KNAT2* probe was added in lane 3 and 4, and mutant *KNAT6* probe was added in lane 7 and 8 as competitor.
(TIF)

S9 Fig *ProBRM*:*BRM-GFP* rescues *brm-1* and *brm-3* phenotype defects. (TIF)

S10 Fig BRM directly bound the *ABI5* promoter. (A) Schematic diagram of *ABI5* for ChIP-qPCR analysis. *e1* (-918 bp to -817 bp) was the loci tested; gray box, 5’ or 3’ untranslated region; black box, exon; gray line, intergenic region. (B) ChIP-qPCR analysis of BRM-GFP DNA fragments co-immunoprecipitated with the anti-GFP antibody. *TA3* and *TUB2* were used as negative control. The values are shown as means±SD.
(TIF)

S1 Table Primers used in this study. (DOCX)
