# Supplementary figures and images for: Arabidopsis BREVIPEDICELLUS Interacts with the SWI2/SNF2 Chromatin Remodeling ATPase BRAHMA to Regulate KNAT2 and KNAT6 Expression in Control of Inflorescence Architecture
Source: PLoS Genet. 2015 Mar 30;11(3):e1005125. doi: 10.1371/journal.pgen.1005125 (PMC4379049; doi:10.1371/journal.pgen.1005125)

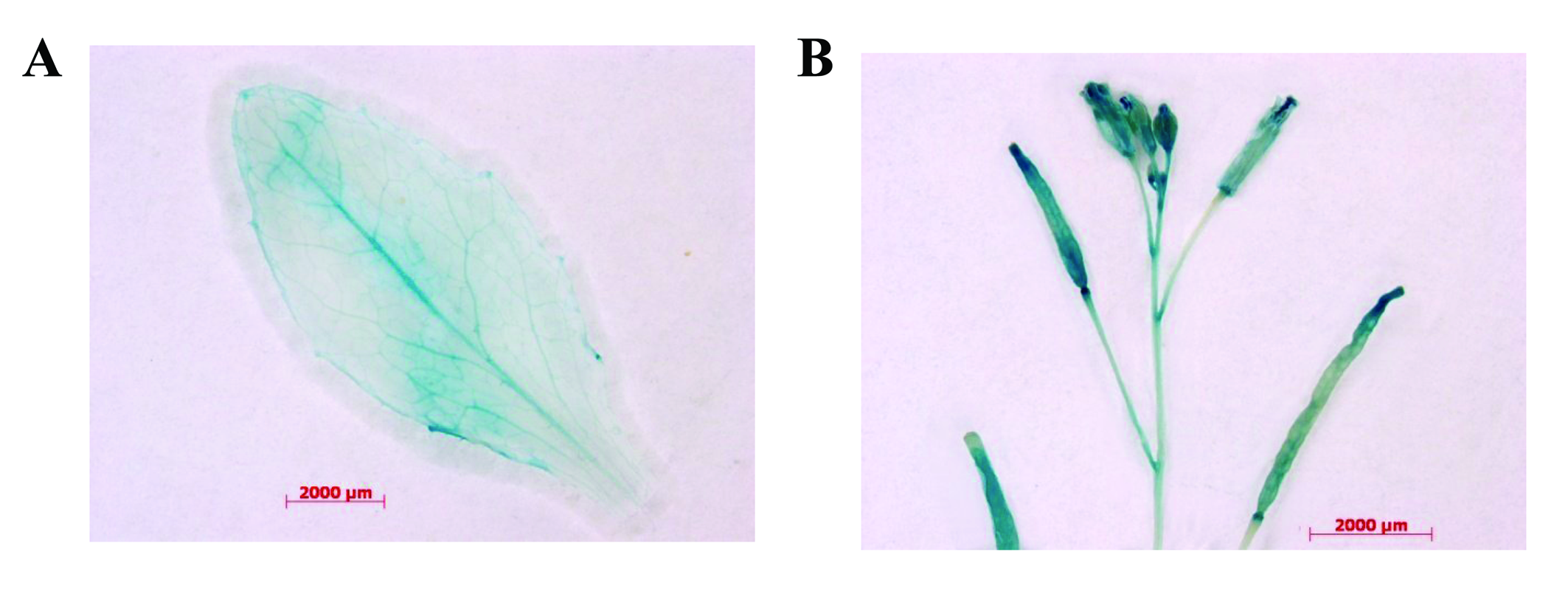

Supplement: S1 Fig — (A) GUS staining of BRM promoter: GUS (pBRM:GUS) observed in the leaf vascular tissues. (B) GUS staining of pBRM:GUS observed in inflorescences. (TIF) [file pgen.1005125.s001.tif]

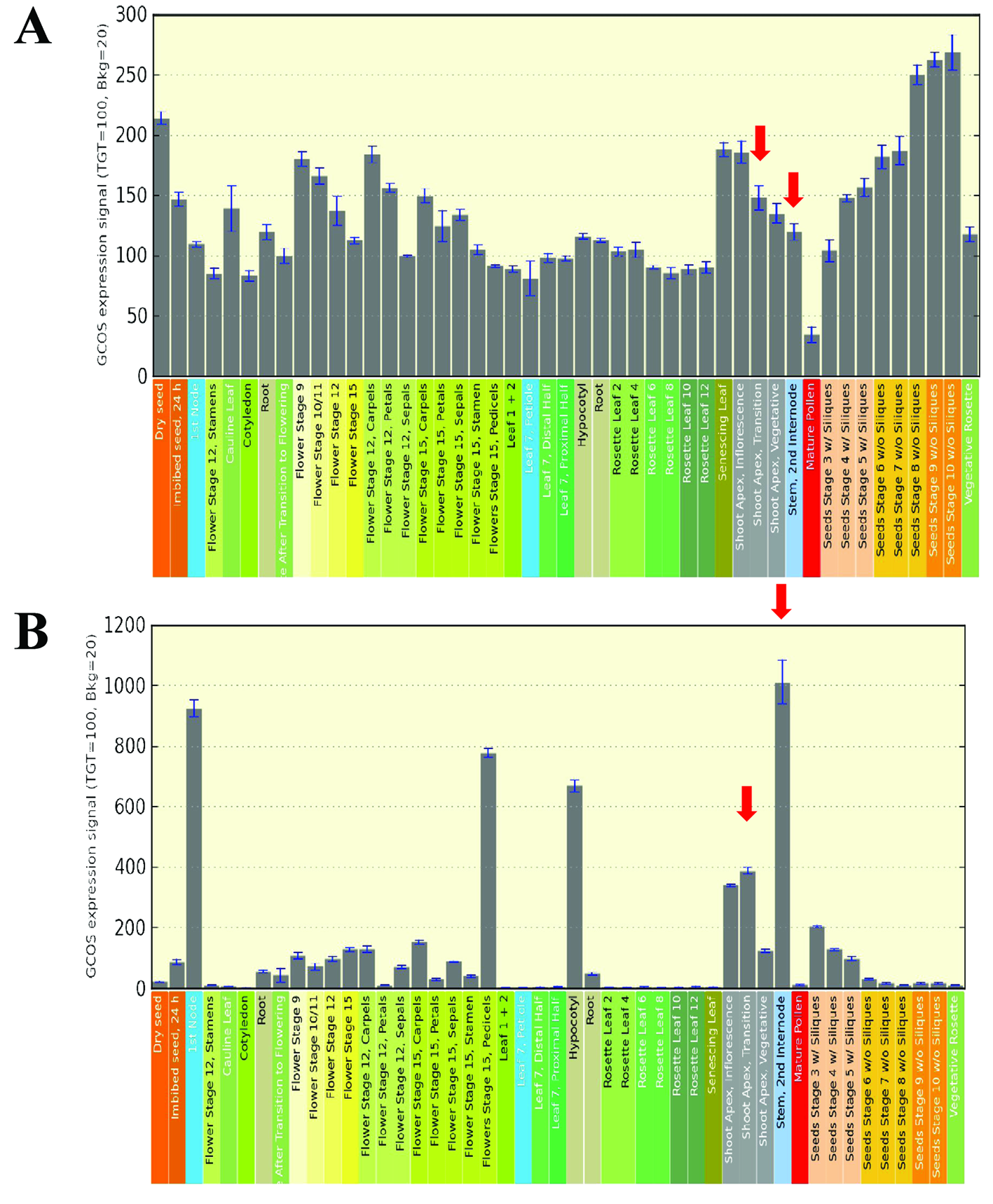

Supplement: S2 Fig — Red arrows indicate the expression levels in shoot apex, stems and internodes. (TIF) [file pgen.1005125.s002.tif]

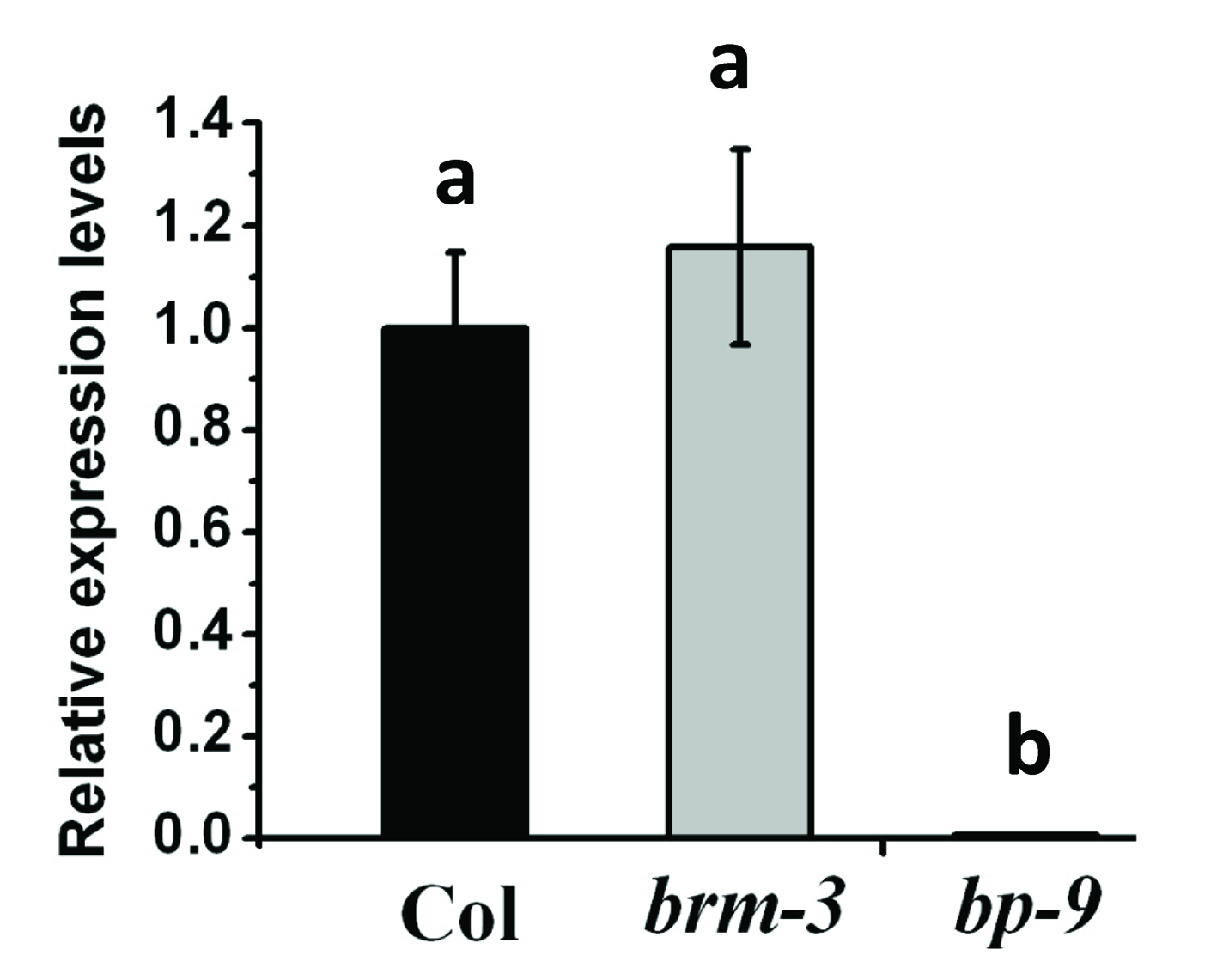

Supplement: S3 Fig — Data shown are means±SD. UBQ was used as an internal control. One-way ANOVA (Tukey-Kramer test) was performed, and statistically significant differences (P < 0.01) are indicated by different lowercase letters (a, b). Equivalent means have the same letter; different letters indicate statistically significant differences. (TIF) [file pgen.1005125.s003.tif]

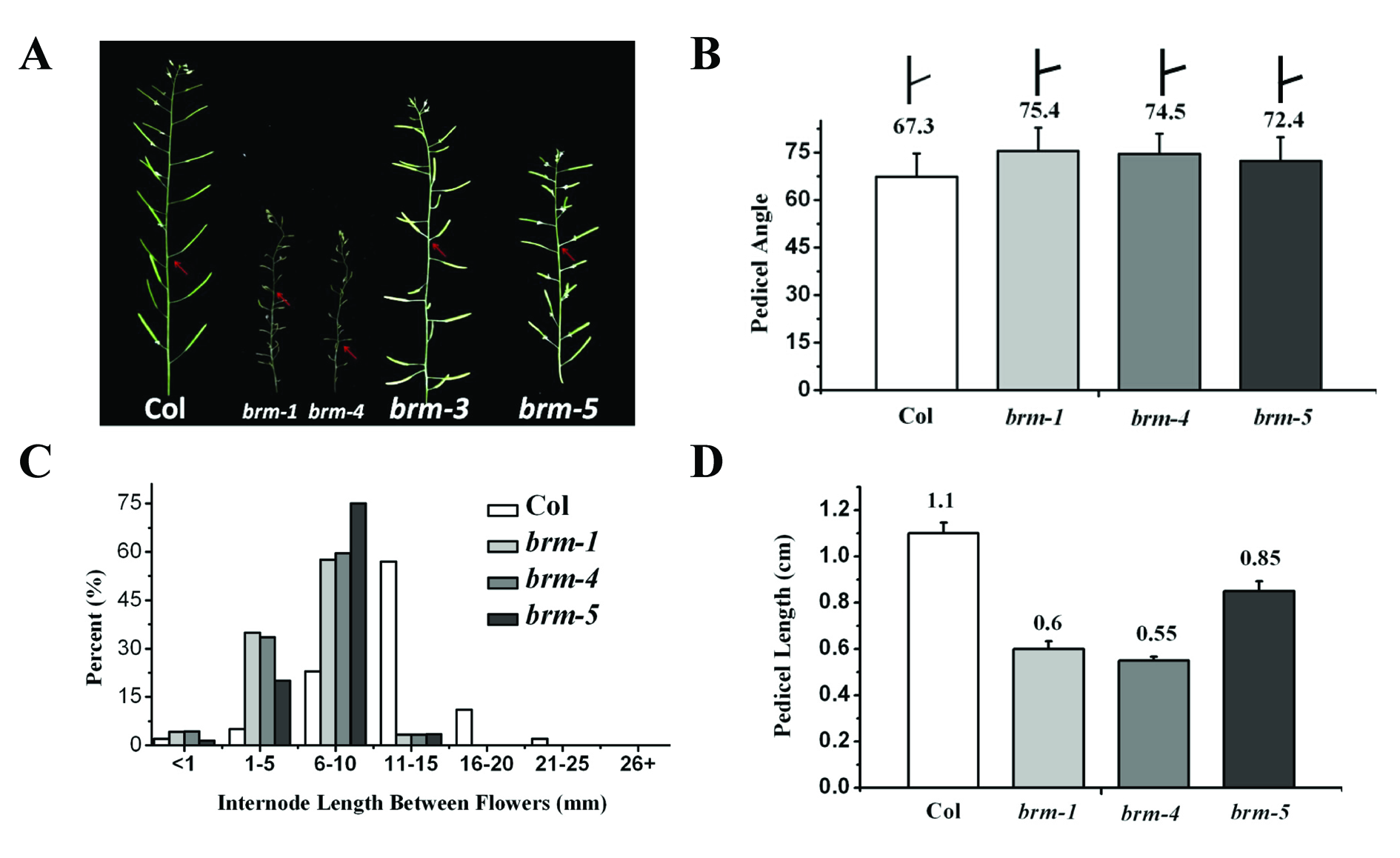

Supplement: S4 Fig — (A) Phenotypes of brm-1, brm-4 and brm-5 mutants. The red color arrows indicate the typical pedicel orientation and internode length of the mutants. (B) Quantitative analysis of the pedicel orientation of brm-1, brm-4 and brm-5 mutants. (C) Distribution of the internode length between two successive siliques in Col, brm-1, brm-4 and brm-5 mutants. Ten internodes between the 1st and 11th siliques were analyzed. (D) Quantitative analysis of the pedicle length of mature siliques. 35-day-old plant were analyzed. (TIF) [file pgen.1005125.s004.tif]

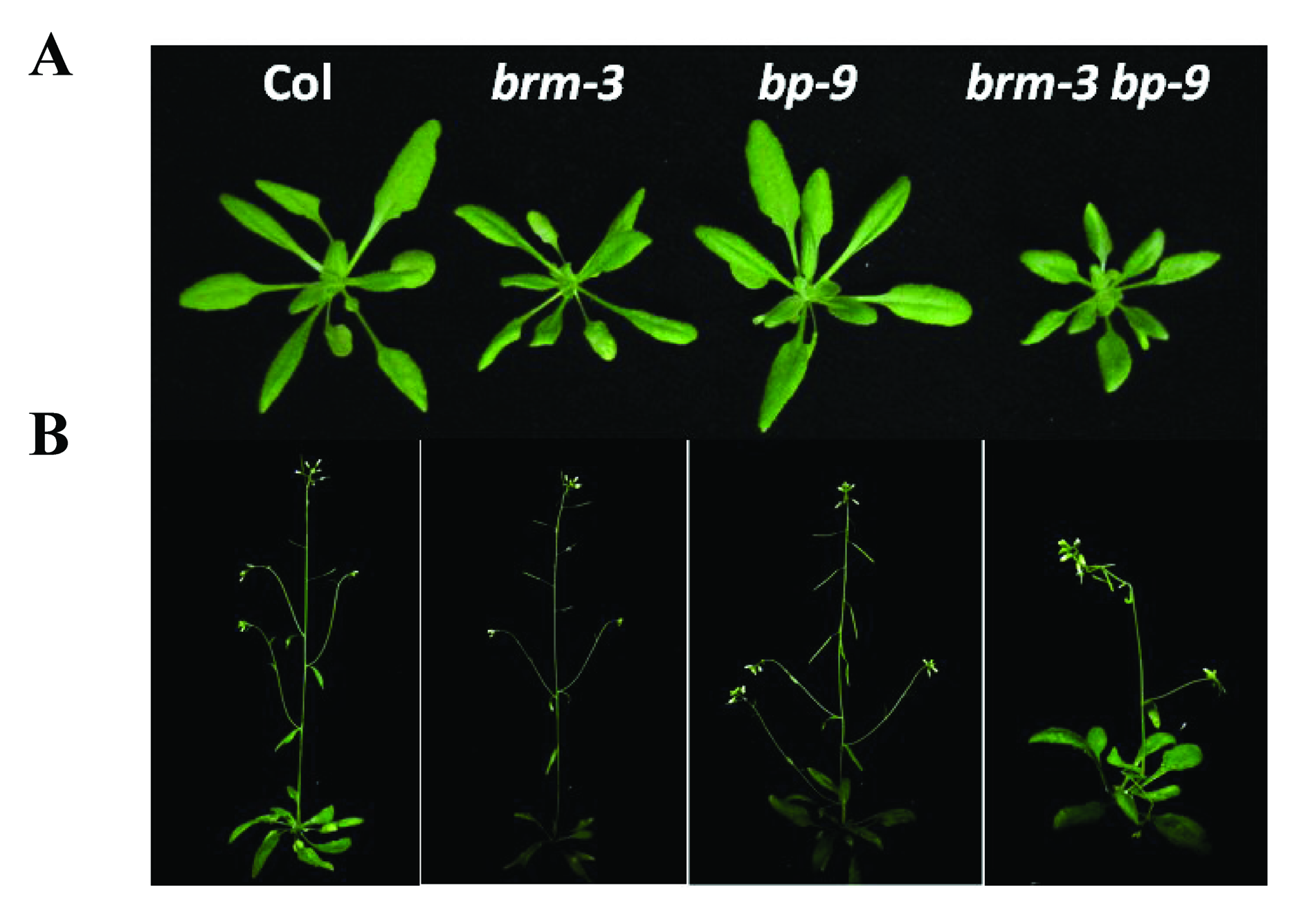

Supplement: S5 Fig — (A) 20-d-ld plants of Col, brm-3, bp-9 and brm-3 bp-9 during vegetative growth. (B) 40-d-old plants of Col, brm-3, bp-9 and brm-3 bp-9. (TIF) [file pgen.1005125.s005.tif]

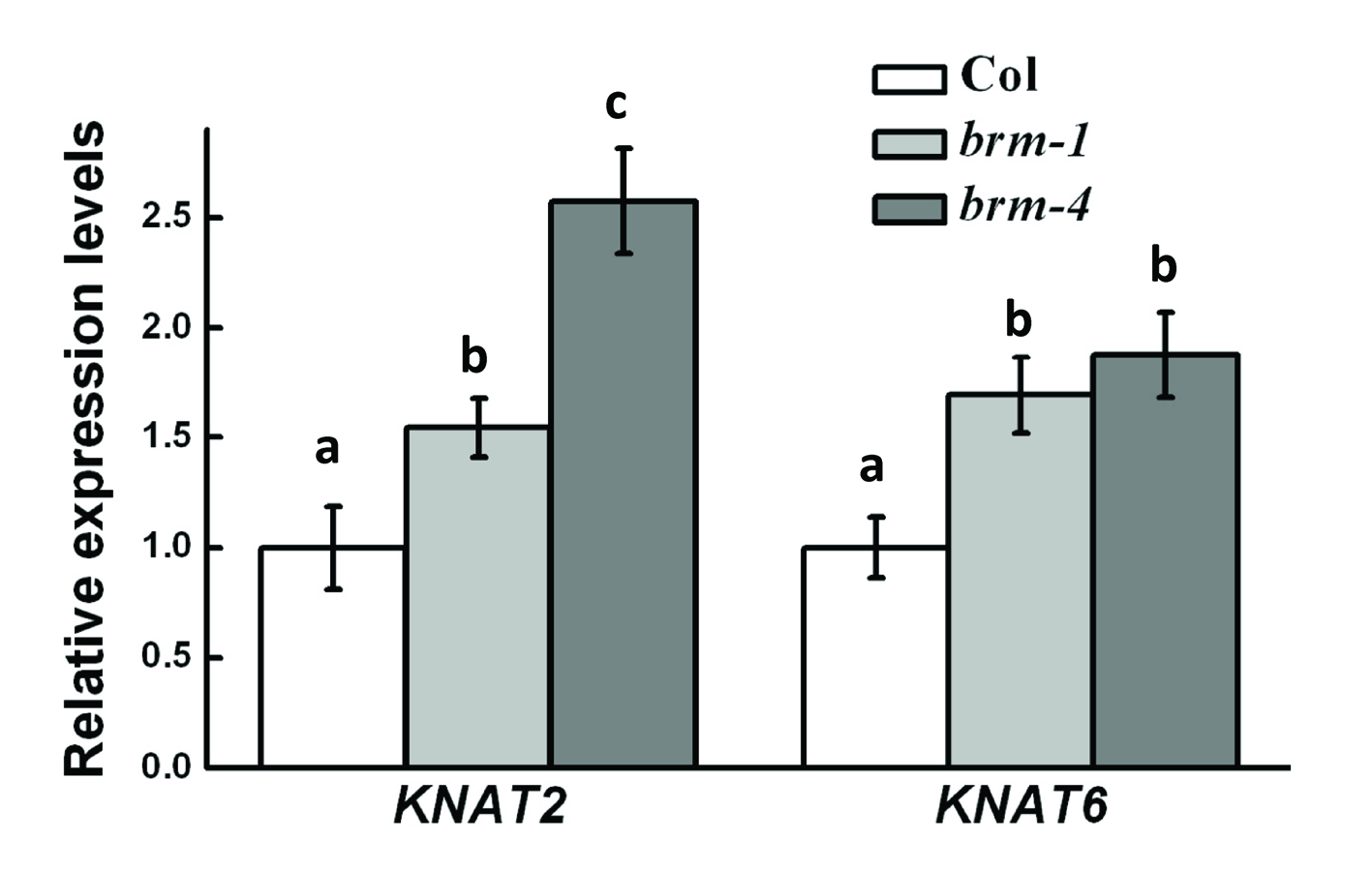

Supplement: S6 Fig — Data shown are means±SD. UBQ was used as an internal control. One-way ANOVA (Tukey-Kramer test) was performed, and statistically significant differences (P < 0.01) are indicated by different lowercase letters (a, b). Equivalent means have the same letter; different letters indicate statistically significant differences. (TIF) [file pgen.1005125.s006.tif]

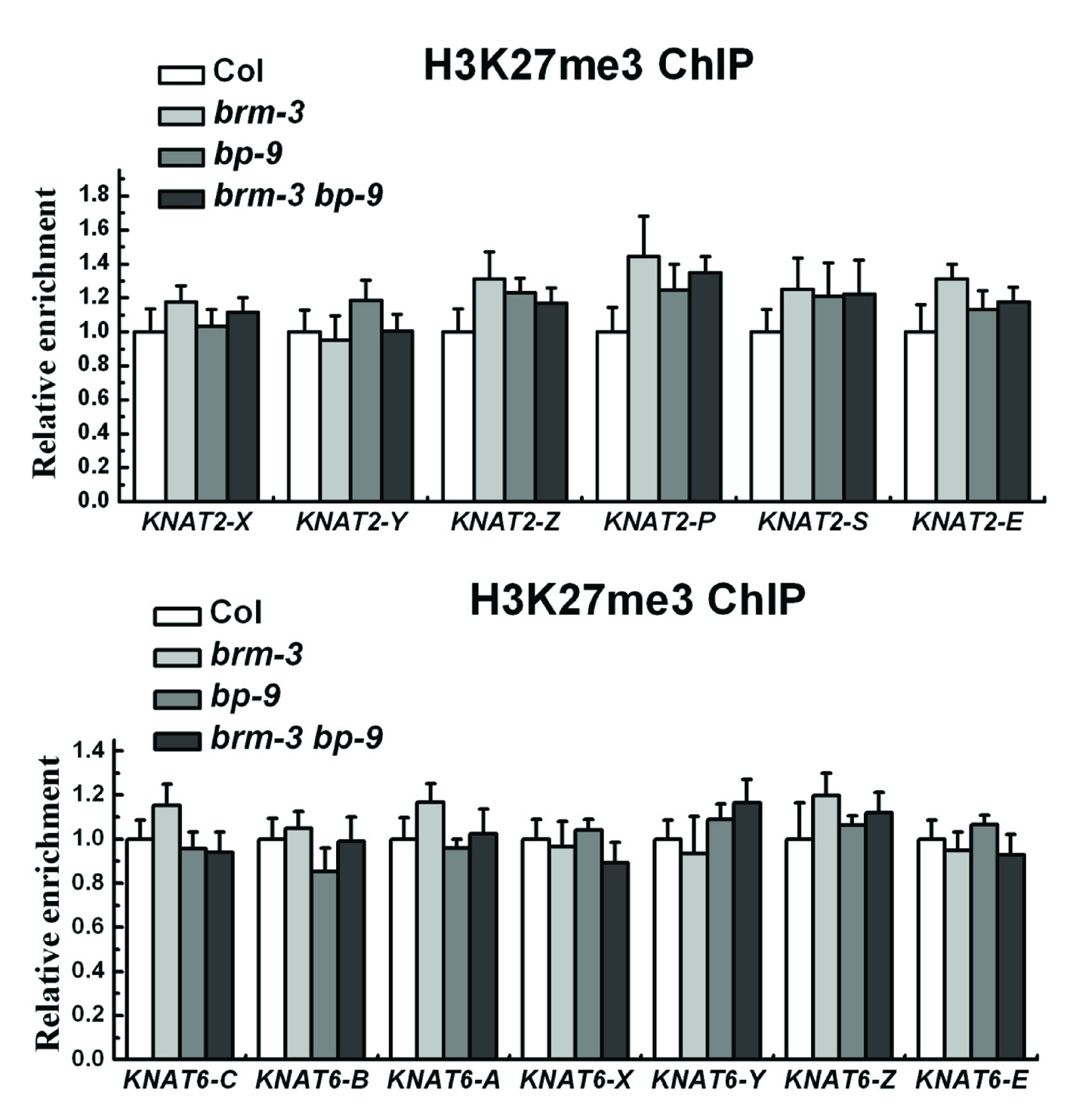

Supplement: S7 Fig — The amounts of DNA after ChIP were quantified and normalized to TUB2. The relative enrichment refers to the H3K27me3 enrichment versus the histone H3 occupancy. The values are shown as means±SD, a single asterisk indicate significant differences from Col by Student’s t test (*P < 0.05). 35-day-old plants were used for analysis. The position of the primers are as indicated in Fig. 6A. (TIF) [file pgen.1005125.s007.tif]

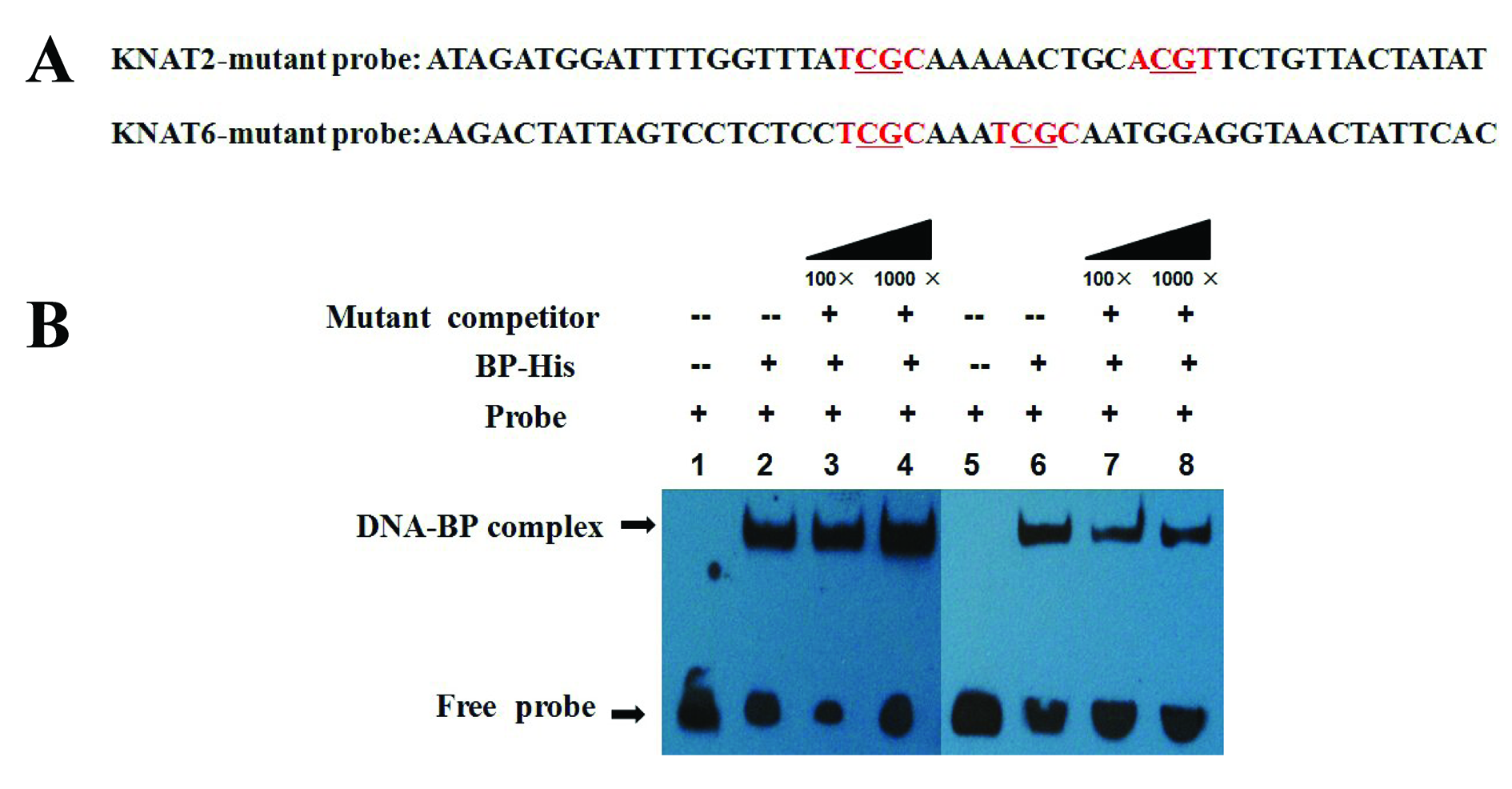

Supplement: S8 Fig — (A) Biotin–labeled mutated probe sequences of KNAT2 (-1039 to -991 bp) and KNAT6 (4269 to 4319 bp). The core binding sites were mutated as shown with red underline. (B) EMSA assay using purified BP-His fusion protein. Lane 1, 2, 3 and 4 were added with KNAT2 probe (20 fmol), whereas lane 5, 6, 7 and 8 were added with KNAT6 probe (20 fmol). 500 ng of BP-His protein was added in lane 2, 3, 4, 6, 7 and 8, and no protein was added in lane 1 and 5 as negative controls. Mutant KNAT2 probe was added in lane 3 and 4, and mutant KNAT6 probe was added in lane 7 and 8 as competitor. (TIF) [file pgen.1005125.s008.tif]

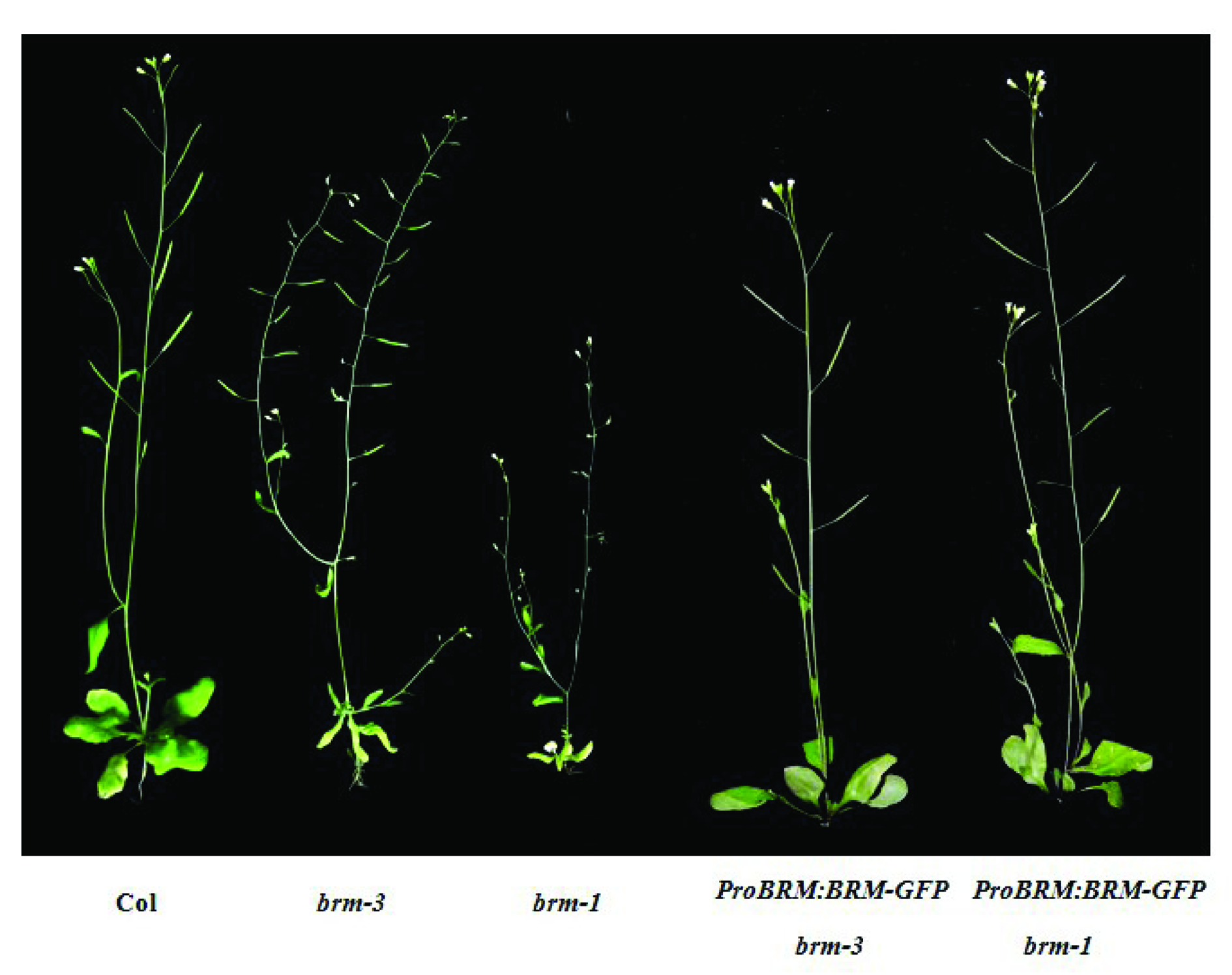

Supplement: S9 Fig — (TIF) [file pgen.1005125.s009.tif]

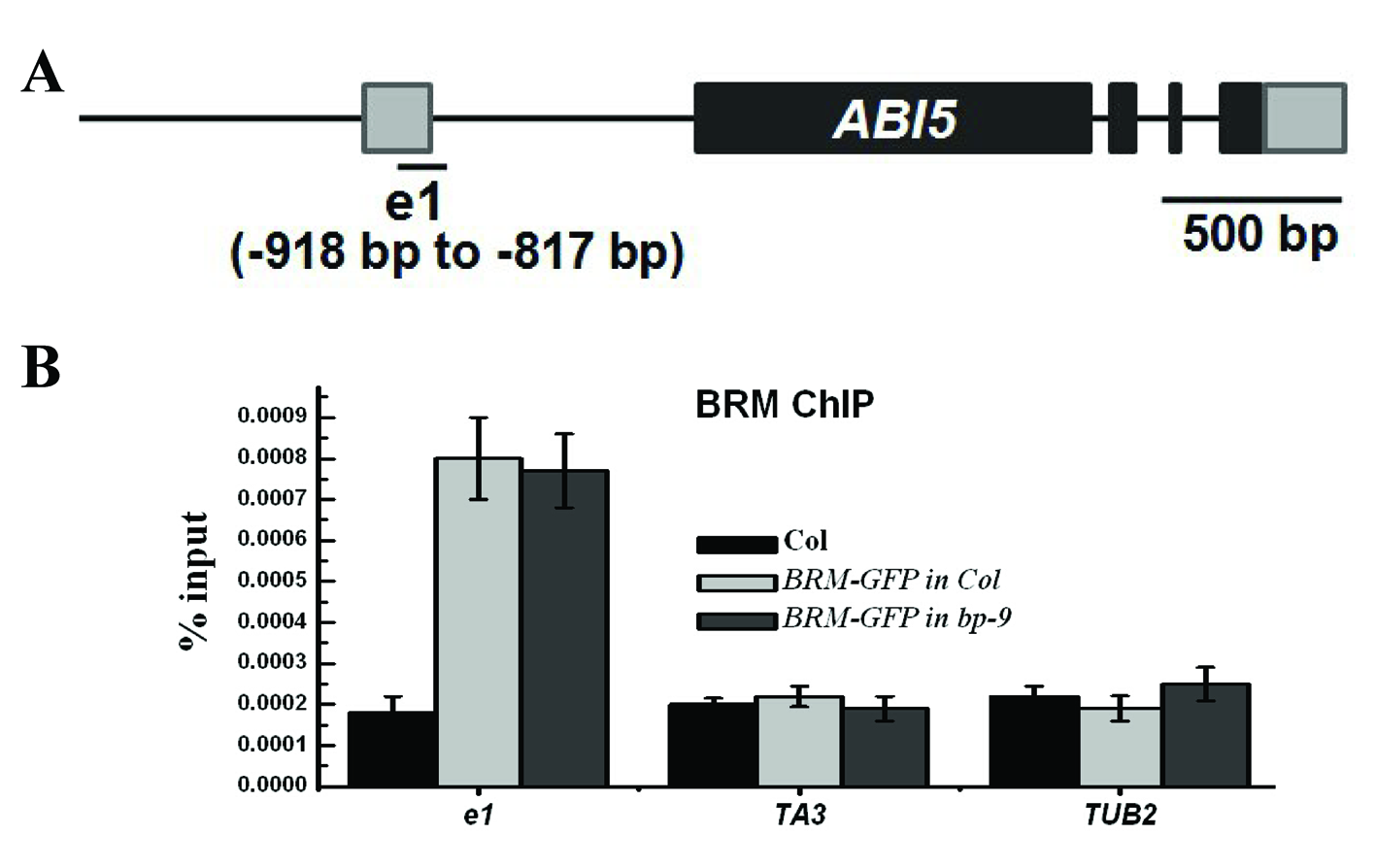

Supplement: S10 Fig — (A) Schematic diagram of ABI5 for ChIP-qPCR analysis. e1 (-918 bp to -817 bp) was the loci tested; gray box, 5’ or 3’ untranslated region; black box, exon; gray line, intergenic region. (B) ChIP-qPCR analysis of BRM-GFP DNA fragments co-immunoprecipitated with the anti-GFP antibody. TA3 and TUB2 were used as negative control. The values are shown as means±SD. (TIF) [file pgen.1005125.s010.tif]
