# Supplementary material for: Arabidopsis BREVIPEDICELLUS Interacts with the SWI2/SNF2 Chromatin Remodeling ATPase BRAHMA to Regulate KNAT2 and KNAT6 Expression in Control of Inflorescence Architecture
Source: PLoS Genet. 2015 Mar 30;11(3):e1005125. doi: 10.1371/journal.pgen.1005125 (PMC4379049; doi:10.1371/journal.pgen.1005125)
Supplement: S1 Table — (DOCX) [file pgen.1005125.s011.docx]

**Suplemental Table 1. Primers used in this study**

**Gene Forward primer (5′-3′) Reverse primer (5′-3′) Purpose**

**BRM-Full** CATGGAGGCCGAATTCATGCAATCTGGAGGCAGTGG GGATCCCCGGGAATTCTAAATGGCTAGGCCGTCTTTTACC Fused to BD

**BRM(1-696aa)** CATGGAGGCCGAATTCATGCAATCTGGAGGCAGTGG GGATCCCCGGGAATTCGAACTCTGCACCTTCTTCACA Fused to BD

**BRM(1-952aa)** CATGGAGGCCGAATTCATGCAATCTGGAGGCAGTGG GGATCCCCGGGAATTCTCTGATCACAACTTCTTCTCGAGC Fused to BD

**BRM(689-952aa)** CATGGAGGCCGAATTCTGTGAAGAAGGTGCAGAGTTC GGATCCCCGGGAATTCTCTGATCACAACTTCTTCTCGAGC Fused to BD

**BRM(953-2193aa)** CATGGAGGCCGAATTCAATAGATTTACGGAAATGAATGCGCC GGATCCCCGGGAATTCTAAATGGCTAGGCCGTCTTTTACC Fused to BD

**BRM-Full** CGATAGTACTGTCGACATGCAATCTGGAGGCAGTGG TACCCTCGAGGTCGACTAAATGGCTAGGCCGTCTTTTACC For BiFC

**BRM-Pro** TGATTACGCCAAGCTTCTTCTACAAACTCGCACACAAGTCA GGGACTGACCTACCCGGGGCAATTGGTCGAAGAATCTGACAG Fused promoter to GUS

**BRM(689-952aa)** GAATTCCCGGGTCGACTGTGAAGAAGGTGCAGAGTTC GGCCGCTCGAGTCGACTCTGATCACAACTTCTTCTCGAGC Fused to GST

**BRM(689-952aa)** GGGACTCTAGAGGATCCTGTGAAGAAGGTGCAGAGTTC TCTGATCACAACTTCTTCTCGAGC Fused to FLAG

**BRM(1576-1877aa)** TCTGATCACAACTTCGCTACTGTTGCTGATTTGTCT GTGGTACCCGGGGATCCGGAAGCTTCAGCGTTATCCT Fused to FLAG

**BP-Full** GGAGGCCAGTGAATTCATGGAAGAATACCAGCATGACAAC CACCCGGGTGGAATTCTGGACCGAGACGATAAGGTC Fused to AD

**BP(1-129aa)** GGAGGCCAGTGAATTCATGGAAGAATACCAGCATGACAAC CACCCGGGTGGAATTCGTTGTCATTGTTGTTGTTGTTGT Fused to AD

**BP(1-240aa)** GGAGGCCAGTGAATTCATGGAAGAATACCAGCATGACAAC CACCCGGGTGGAATTCAATGGGACTCTGACACAGCAT Fused to AD

**BP(130-240aa)** GGAGGCCAGTGAATTCGTAAGCGATGTTGAAGCCATG CACCCGGGTGGAATTCAATGGGACTCTGACACAGCAT Fused to AD

**BP(241-400aa)** GGAGGCCAGTGAATTCCACATCCTCAACAATCCTGAT CACCCGGGTGGAATTCTGGACCGAGACGATAAGGTC Fused to AD

**BP-Full** GGACTCTTGACCATGGATGGAAGAATACCAGCATGACAAC GTCAGATCTACCATGGTTGGACCGAGACGATAAGGTC Fused to YFP

**BP-Full** CGATAGTACTGTCGACATGGAAGAATACCAGCATGACAAC TACCCTCGAGGTCGACTGGACCGAGACGATAAGGTC For BiFC

**BP-Full** TATCGGATCCGAATTCATGGAAGAATACCAGCATGACAAC GACGGAGCTCGAATTCTGGACCGAGACGATAAGGTC Fused to His

**BP-Full** ATCCTCTAGAGTCGACATGGAAGAATACCAGCATGACAAC GTCAGATCTACCATGGTTGGACCGAGACGATAAGGTC For ProBP:BP-GFP construction

**BP-Pro** ATCCTCTAGAGTCGACTATCGTATAATATCCACTATCCTGT ATTCTTCCATGTCGACACCCAGATGAGTAAAGATTTGAGA For ProBP:BP-GFP construction

**BP-Full** GGACTCTTGACCATGGATGGAAGAATACCAGCATGACAAC GTCAGATCTACCATGGTTGGACCGAGACGATAAGGTC For 35S:BP-GFP construction

**BP** GGGAAGAGTGACAATATGGG TATGGACCGAGACGATAAGG For qRT-PCR

**KNAT2** TCATCTGACGAGGAACTGAG CGTCCATCATATCAATCGGC For qRT-PCR

**KNAT6** CTTACTTCAAGCTTACATCGATTGC CGCAGTACGTTTCCATAAATTCATC For qRT-PCR

**KNAT2-X** CCTGAGCTAATTAAGTAGA GGAGCTAATTTTGCTTATG For ChIP qRT-PCR

**KNAT2-Y** CTGTCGTTTTTATAAGGTTTG CACTTATCGCACTTCTTGTTA For ChIP qRT-PCR

**KNAT2-Z** GTGCAACCATTGCTGAAGAGA ACATATCAACGTACCTGTAAGA For ChIP qRT-PCR

**KNAT2-P** AACCGATCCGGTTAGACAAC CTGTACGATTACATGGTTACG For ChIP qRT-PCR

**KNAT2-S** CTTCAGAAGCAAGCACAAGG TTCGTCGGATCCAAACAGTC For ChIP qRT-PCR

**KNAT2-E** AGTTACAGATCTTGCGGATTAG CAGCTACAACAACTTATCGATTC For ChIP qRT-PCR

**KNAT2-M** GGTCAGTATGGTATGGTCAT ACTTACCACTAGCCACCTGA For ChIP qRT-PCR

**KNAT2-N** GCTGCTTTAGCCACTTAGTCA TGTACTAGGAGAATGTATGGTG For ChIP qRT-PCR

**KNAT6-X** GTATGTATCAGCTCACTAGTTG GAAGTCGCATATCAGAAGATA For ChIP qRT-PCR

**KNAT6-Y** TACCATCTAAGTGTATTGTCG AGGTCCAAGGCTGTGTTATGA For ChIP qRT-PCR

**KNAT6-Z** TCTCTCTACACACTCCCATAG GTATAAGATCTCCGGTAAGAAC For ChIP qRT-PCR

**KNAT6-A** TCATCATGAATATCAGCATCATC CTAAAGTAAACACTAGCTGATAC For ChIP qRT-PCR

**KNAT6-B** ACCAAGAATATGCATATGTGAAC GCTCTAAATCACCTAACGTATC For ChIP qRT-PCR

**KNAT6-C** GATGGGTCAATCAACCTGTC ATGAGTTTAGTGCTTCGAAAC For ChIP qRT-PCR

**KNAT6-E** GTGGTTAGTTCTGAATGATT CTATCAACAAGCTACCAATC For ChIP qRT-PCR

**KNAT6-M** TGACTAAGCACGGTTGCTTG ACGACTCTACTGACCCACTTA For ChIP qRT-PCR

**KNAT6-N** CTCTTTAGCCACTTAGCCGT GACATCAAAGACAGAGAATGGT For ChIP qRT-PCR

**UBQ** GATCTTTGCCGGAAAACAATTGGAGGATGGT CGACTTGTCATTAGAAAGAAAGAGATAACAGG For qRT-PCR

**TUB2** ACAAACACAGAGAGGAGTGAGCA ACGCATCTTCGGTTGGATGAGTGA For ChIP qRT-PCR

**TA3** CTGCGTGGAAGTCTGTCAAA CTATGCCACAGGGCAGTTTT For ChIP qRT-PCR
